# Water/fat separation for self‐navigated diffusion‐weighted multishot echo‐planar imaging

**DOI:** 10.1002/nbm.4822

**Published:** 2022-09-13

**Authors:** Yiming Dong, Malte Riedel, Kirsten Koolstra, Matthias J. P. van Osch, Peter Börnert

**Affiliations:** ^1^ C. J. Gorter Center for High Field MRI, Department of Radiology Leiden University Medical Center Leiden The Netherlands; ^2^ Institute for Biomedical Engineering ETH Zurich and University of Zurich Zurich Switzerland; ^3^ Division of Image Processing, Department of Radiology Leiden University Medical Center Leiden The Netherlands; ^4^ Philips Research Hamburg Hamburg Germany

**Keywords:** chemical shift encoding, diffusion, multishot EPI, MUSE, self‐navigation

## Abstract

The purpose of this study was to develop a self‐navigation strategy to improve scan efficiency and image quality of water/fat‐separated, diffusion‐weighted multishot echo‐planar imaging (ms‐EPI). This is accomplished by acquiring chemical shift‐encoded diffusion‐weighted data and using an appropriate water‐fat and diffusion‐encoded signal model to enable reconstruction directly from k‐space data. Multishot EPI provides reduced geometric distortion and improved signal‐to‐noise ratio in diffusion‐weighted imaging compared with single‐shot approaches. Multishot acquisitions require corrections for physiological motion‐induced shot‐to‐shot phase errors using either extra navigators or self‐navigation principles. In addition, proper fat suppression is important, especially in regions with large B_0_ inhomogeneity. This makes the use of chemical shift encoding attractive. However, when combined with ms‐EPI, shot‐to‐shot phase navigation can be challenging because of the spatial displacement of fat signals along the phase‐encoding direction. In this work, a new model‐based, self‐navigated water/fat separation reconstruction algorithm is proposed. Experiments in legs and in the head–neck region of 10 subjects were performed to validate the algorithm. The results are compared with an image‐based, two‐dimensional (2D) navigated water/fat separation approach for ms‐EPI and with a conventional fat saturation approach. Compared with the 2D navigated method, the use of self‐navigation reduced the shot duration time by 30%–35%. The proposed algorithm provided improved diffusion‐weighted water images in both leg and head–neck regions compared with the 2D navigator‐based approach. The proposed algorithm also produced better fat suppression compared with the conventional fat saturation technique in the B_0_ inhomogeneous regions. In conclusion, the proposed self‐navigated reconstruction algorithm can produce superior water‐only diffusion‐weighted EPI images with less artefacts compared with the existing methods.

## Graphical abstract

A model‐based, self‐navigated water/fat decomposition algorithm (MSND) for diffusion‐weighted multishot EPI is proposed in this work. This is achieved by jointly estimating physiological motion‐induced shot‐to‐shot phase variations while performing water/fat separation using chemical shift encoding. In vivo examples demonstrate improved water/fat separation when using self‐navigation to eliminate the shot‐to‐shot phase errors.

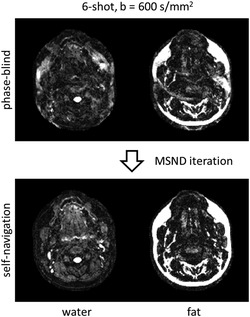

**Abbreviations**
2D: two‐dimensional
ADC: apparent diffusion coefficient
CG: conjugate gradient
CoV: coefficient of variation
CSM: coil‐sensitivity map
DWI: diffusion‐weighted imaging
EPI: echo‐planar imaging
ESPIRiT: Eigenvector‐based iTerative Self‐consistent Parallel Imaging Reconstruction
FFE: fast field echo (gradient echo)
IDE: image‐based, water/fat decomposition approach for EPI
J‐SENSE: joint image reconstruction and sensitivity estimation in SENSE
ms‐EPI: multishot echo‐planar imaging
MSND: Model‐based Self‐Navigated water/fat Decomposition
MUSE: multiplexed sensitivity‐encoding
SENSE: sensitivity encoding for fast MRI
SNR: signal‐to‐noise ratio
SPAIR: SPectral Attenuated Inversion Recovery
SPIR: Spectral Presaturation with Inversion Recovery
ss‐EPI: single‐shot echo‐planar imaging
TV: total‐variation

## 1 INTRODUCTION

Over the years, diffusion‐weighted imaging (DWI) has been widely used to detect and characterize different pathologies by measuring the movement and transport of water molecules.^1^, ^2^, ^3^ Traditionally, single‐shot echo‐planar imaging (ss‐EPI) has been used as the standard readout for clinical diffusion scans because of its ability to freeze physiological motion effects. However, the in‐plane resolution of ss‐EPI is limited and the low bandwidth along the phase‐encoding direction causes significant geometric distortions in regions with large B_0_ inhomogeneities.^4^ To mitigate these effects, several multishot EPI (ms‐EPI)^5^ readout approaches have been proposed, achieving higher image resolution and better signal‐to‐noise ratio (SNR).^6^, ^7^

A prime challenge for DW ms‐EPI is dealing with shot‐to‐shot phase variations (diffusion phases).^8^, ^9^, ^10^ These phase changes arise mainly from physiological motion effects (e.g., cardiac pulsation and respiration) and its interplay with the strong diffusion sensitizing gradients that cause differences for each shot.^7^, ^8^, ^9^ Application of standard reconstruction approaches to ms‐EPI DWI may yield nondiagnostic images as a result of such shot‐to‐shot phase inconsistencies.^11^ Recent studies have introduced several approaches to address shot‐to‐shot phase errors using (1) measured extra navigators,^7^, ^12^, ^13^ (2) self‐navigation, by directly estimating the phase variations among different shots from the imaging data,^11^, ^14^, ^15^, ^16^, ^17^, ^18^ and/or (3) navigator‐free reconstructions, by applying low‐rank constraints.^19^, ^20^, ^21^ Most of these ms‐EPI–based DWI studies focused on brain images. However, the use of diffusion MRI in other parts of the body is also well established, such as tumor characterization and treatment monitoring in the head–neck region.^2^, ^22^ Also, here shot‐to‐shot phase errors are a problem that requires special correction methods.

However, when employing DW EPI, fat becomes a serious confounding factor because of the potential failure of conventional fat suppression techniques (e.g., Spectral Presaturation with Inversion Recovery [SPIR]/SPectral Attenuated Inversion Recovery [SPAIR]^23^, ^24^) in regions of inhomogeneous B_0._ ^25^, ^26^, ^27^ In particular, when using EPI, the large chemical shift will lead to a significant spatial displacement of the fat signal along the phase‐encoding direction. Consequently, the shifted fat signal, which exhibits only minor diffusion attenuation, may overlap with important water structures and can compromise clinical diagnosis.^28^, ^29^, ^30^, ^31^, ^32^ In addition, standard spectrally selective fat saturation methods are shown to be unable to suppress minor fat resonances of the multipeak fat spectrum^33^, ^34^ that are close to the water resonance frequency.^29^, ^30^, ^32^ All these factors led to growing interest in applying chemical shift encoding^35^, ^36^ to DWI to achieve sufficient water/fat separation. For DW EPI, researchers proposed either using only chemical shift encoding to handle aspects of the multipeak fat spectrum^31^, ^32^ or combining chemical shift encoding with spectrally selective fat‐suppression techniques.^29^, ^30^

In our previous study, a two‐step reconstruction framework (an image‐based, water/fat decomposition approach for EPI [IDE]^32^) was employed. Multiple ms‐EPI images acquired at differently shifted echo times (TEs) were used to achieve chemical shift encoding.^37^ The information from a two‐dimensional (2D) navigator^7^ was used to reconstruct the chemical shift‐encoded source images, followed by an image‐based water/fat separation with intrinsic B_0_ map estimation.^32^ In the method of Hu et al.,^31^ both B_0_ and fat off‐resonance effects were corrected by measuring an additional point spread function (PSF) dimension,^38^, ^39^ allowing to also correct for geometric distortions. However, for both methods, the shifted fat in the measured extra 2D navigator is ignored, which might compromise the shot‐wise phase estimation. This may lead to artifacts in the final water image, especially in areas where water and shifted fat overlap. Moreover, the fidelity of the phase estimates and thus the quality of the reconstrued DW images may suffer from the poor SNR of the extra navigators acquired at TEs often larger than 100 ms. Furthermore, the use of the additional extra navigator is accompanied by a significant drop in scan efficiency, thereby prolonging the scanning time by approximately 30%–50%.^17^

In this work, we propose a new reconstruction approach aimed at improving the quality of DW images by removing their fat signals and enhancing the acquisition efficiency of chemical shift‐encoded DW ms‐EPI by alleviating the need for extra 2D navigator measurements. We name this new approach “Model‐based Self‐Navigated water/fat Decomposition” (MSND). The method enables the joint calculation of water and fat components while estimating shot‐specific phase maps directly from the DW raw data for each b‐value. In vivo validation in the leg and head–neck region show that the proposed MSND algorithm can improve the image quality compared with previous approaches. The fat‐suppression quality of the MSND method is also demonstrated in B_0_ inhomogeneous regions and is shown to outperform conventional fat saturation (SPIR/SPAIR).

## 2 THEORY

Chemical shift encoding and self‐navigation in ms‐EPI–based DWI are two distinct aspects, which are dealing with different phase contributions. To estimate and combine them simultaneously in a reconstruction pipeline that ultimately solves for water and fat, the B_0_ off‐resonance and the motion‐induced shot‐to‐shot phase information need to be carefully resolved in the reconstruction. Furthermore, a potential mismatch between sensitivity encoding (coil sensitivities) and Fourier encoding (EPI sampling and its off‐resonance behavior) must be resolved to ensure data consistency. To address the various elements of the corresponding reconstruction pipeline, the Theory section is organized as follows:

1. An extended model is introduced, considering the k‐space data and all relevant parameters.
2. Water/fat separation based on (1) is introduced, aimed at eliminating the spatial displacement and mismatch between water and fat components caused by chemical shift during EPI sampling.
3. Self‐navigation is introduced to eliminate the phase variations between shots, using a Gauss‐Newton loop estimating the shot‐specific phase maps.
4. Furthermore, to better steer the overall convergence of the joint algorithm that is addressing (2) and (3) above, a multiplexed sensitivity‐encoding (MUSE)^15^‐like initialization step is proposed.
5. A geometric distortion‐adapted coil sensitivity map calibration is performed, as a preparatory step, to mitigate the misalignment between coil sensitivity maps (CSMs) and EPI data caused by spatial off‐resonance effects.

These aspects will be detailed in the following, before combining them into a reconstruction pipeline in section 3.2.

### 2.1 Extended signal model

**FIGURE 1:**
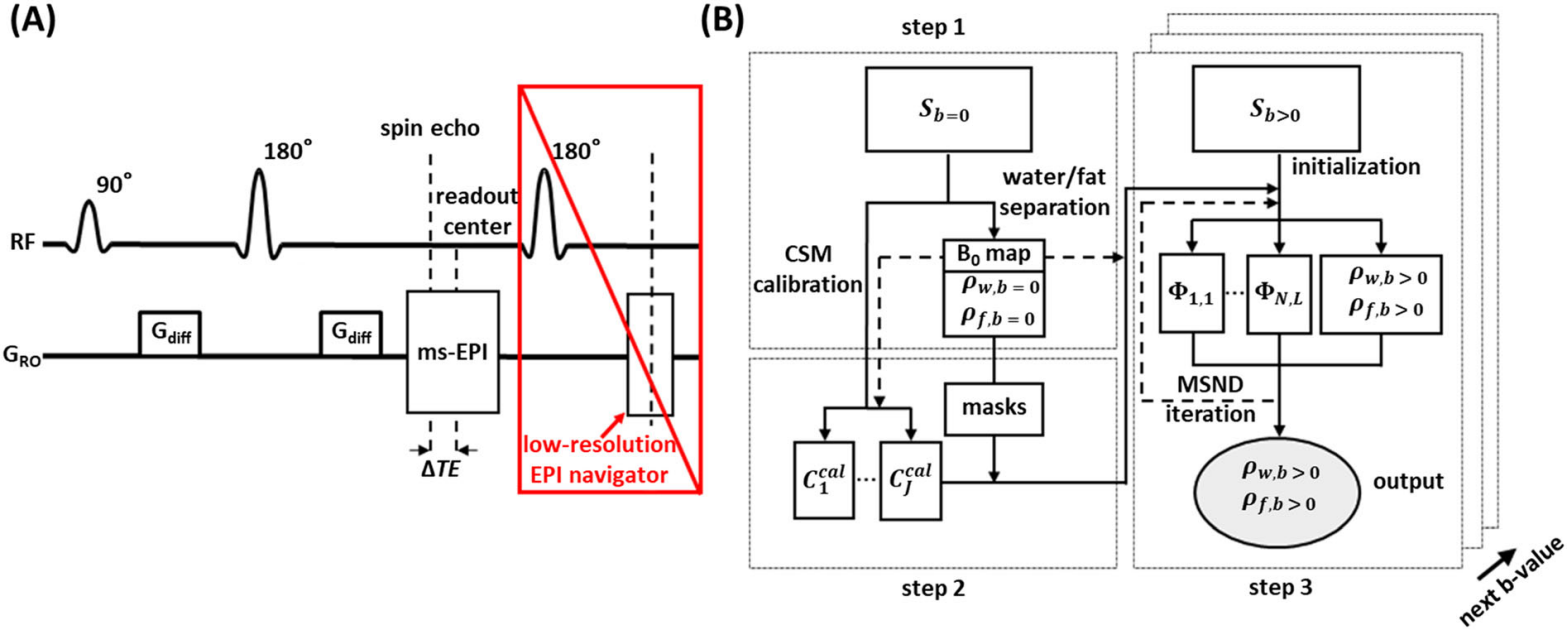
Simplified sequence diagram and the reconstruction flowchart. (A) Chemical shift‐encoded, diffusion‐weighted (DW), multishot echo‐planar imaging (ms‐EPI) sequence equipped with an extra 2D low‐resolution, single‐shot EPI navigator for comparison. Chemical shift encoding is enabled by shifting the readout window back and forth ( ΔTE). In this work, one objective is to eliminate the measured extra navigator (red box). (B) Reconstruction pipeline assuming a DWI dataset with N chemical shift‐encoding steps, L shot, J coils, and with multiple b‐values (b = 0 s/mm^2^ and b > 0 s/mm^2^). The reconstruction includes the following three steps: (1) performing water/fat separation on nondiffusion data $S_{b=0}$ to calculate separated water/fat images $P_{w,b=0}$/ $P_{f,b=0}$. In this work, the IDE^32^ algorithm is used to separate water/fat, calculating also a B_0_ map that is used in the coil‐sensitivity maps (CSMs) calibration and Model‐based Self‐Navigated water/fat Decomposition (MSND) algorithm; (2) calibrating the CSMs using b = 0 s/mm^2^ data, then masking each calibrated CSM ( $C_{j}^{\mathrm{cal}}$) with threshold water/fat masks‐calculated images $P_{w,b=0}$/ $P_{f,b=0}$; and (3) reconstructing the DW data $S_{b>0}$ using the proposed MSND algorithms with reasonable initialization to estimate the motion‐induced shot‐to‐shot phase variations $\Phi_{n,l}$ and DW water/fat images $P_{w,b>0}$/ $P_{f,b>0}$ for each b‐value

In a chemical shift‐encoded DW ms‐EPI sequence, the acquisition is repeated N times (often N = 3) at different $\Delta {\mathrm{TE}}_{n}$ to encode water and fat signals. The $\Delta {\mathrm{TE}}_{n}$ is defined by the spacing between the center of the EPI readout window and the center of the actual spin‐echo and can be attained by shifting the sampling window back and forth, as illustrated in Figure 1A. The complex ms‐EPI signal $s_{n,l,j}\left(t\right)$ for a k‐space sample $k_{t}$ at time t, shot l, coil j, and chemical shift‐encoding point n can be written as

(1) $$s_{n,l,j}\left(t\right)=\int \left[c_{j}\left(r\right)\rho_{w}\left(r\right)+\sum_{m=1}^{M}\alpha_{m}c_{j}\left(r\right)\rho_{f}\left(r\right)e^{-i2\pi \psi_{f,m}\left(\Delta {\mathrm{TE}}_{n}+t\right)}\right]e^{-i2\pi \psi_{B}\left(r\right)\Delta {\mathrm{TE}}_{n}}e^{-i\phi_{n,l}\left(r\right)}e^{-ik_{t}\cdot r}\mathrm{dr},$$

where $\rho_{w}$ and $\rho_{f}$ are the complex‐valued DW water/fat components for a given b‐value, $c_{j}$ denotes the coil sensitivities, $\alpha_{m}$ and $\psi_{f,m}$ (in Hz) are the relative amplitude and chemical shift for each peak m of the M‐peak fat model, and r indicates the spatial position. $\psi_{B}$ and $\phi_{n,l}$ denote the B_0_ inhomogeneity‐ (in Hz) and motion‐induced phase map. Note that in this model the B_0_‐induced dephasing during the readout process is ignored by assuming $e^{-i2\pi \psi_{B}\left(r\right)t}\approx e^{-i2\pi \psi_{B}\left(r\right)\Delta {\mathrm{TE}}_{n}}$.

### 2.2 Water fat separation

To solve the linear system corresponding to Equation 1 for water and fat appropriately, the raw data $s_{n,l,j}$ were linked to the water/fat‐separated images $\rho_{w}$ and $\rho_{f}$ via linear operators. Because the chemical shift of fat is spatially invariant, the order of the fat phase modulation term $\alpha_{m}e^{-i2\pi \psi_{f,m}\left(\Delta {\mathrm{TE}}_{n}+t\right)}$ and the integral in Equation 1 can be reversed, that is,

(2) $$\begin{array}{c} \int \left[\sum_{m=1}^{M}\alpha_{m}\;\rho_{f}\left(r\right)e^{-i2\pi \psi_{f,m}\left(\Delta {\mathrm{TE}}_{n}+t\right)}\right]e^{-ik_{t}\cdot r}\mathrm{dr}=\sum_{m=1}^{M}\alpha_{m}e^{-i2\pi \psi_{f,m}\left(\Delta {\mathrm{TE}}_{n}+t\right)}\int \rho_{f}\left(r\right)e^{-ik_{t}\cdot r}\mathrm{dr} \end{array}$$

dropping coil sensitivities, B_0_ inhomogeneity‐ and motion‐induced phase maps for the purposes of illustration. This allows to simulate the fat off‐resonance effect via a weighted Fourier transform. Thus the total signal model for a certain b‐value with N chemical shift‐encoding steps, *L* shots, and *J* coils can be expressed as:

(3) $$S=\hat{K}\left[\;\hat{I}\;\hat{I}\right]\left[\;\begin{array}{c} \hat{F}\\ 0 \end{array}\;\begin{array}{c} 0\\ {\hat{\Psi }}_{f}\hat{F} \end{array}\;\right]\left[\;\begin{array}{c} \hat{C}\\ 0 \end{array}\;\begin{array}{c} 0\\ \hat{C} \end{array}\;\right]\left[\;\begin{array}{c} {\hat{\Psi }}_{B}\\ 0 \end{array}\;\begin{array}{c} 0\\ {\hat{\Psi }}_{B} \end{array}\;\right]\left[\;\begin{array}{c} \hat{\Phi }\\ 0 \end{array}\;\begin{array}{c} 0\\ \hat{\Phi } \end{array}\;\right]\left[\;\begin{array}{c} {Ρ}_{w}\\ {Ρ}_{f} \end{array}\right]=\hat{A}X,$$

where $X={\left[{Ρ}_{w},{Ρ}_{f}\right]}^{T}={\left[{\rho_{w}}^{1},\ldots ,{\rho_{w}}^{Q},{\rho_{f}}^{1},\ldots ,{\rho_{f}}^{Q}\right]}^{T}$ is the target water/fat image with total number of voxels Q. The linear operator $\hat{\Phi }$ describes the motion‐induced diffusion phase, ${\hat{\Psi }}_{B}$ the B_0_ off‐resonance–induced phase, and ${\hat{\Psi }}_{f}$ the fat off‐resonance–induced phase. $\hat{C}$ performs the coil sensitivity weighting,^40^ $\hat{F}$ is the Fourier transform, and $\;\hat{I}$ the identity matrix. $\hat{K}$ is the sampling operator constructed from the k‐space trajectory of each shot. $\hat{A}$ is the total system matrix containing all the above‐defined operators. More details about the construction of each operator can be found in the supporting information (S.1).

Equation 3 can be solved as a least‐squares problem by minimizing:

(4) $$\begin{array}{c} {\left\{{Ρ}_{w},{Ρ}_{f}\right\}}^{*}=\underset{{Ρ}_{w},{Ρ}_{f}\in {\mathbb{C}}^{Q}}{\text{argmin}}{\left\|\hat{A}X-S\right\|}_{2}^{2}. \end{array}$$

### 2.3 Self‐navigation: Shot‐to‐shot phase estimation

Solving Equation 4 requires knowledge of the motion‐induced phase maps for each shot. In general, it is assumed that for a given voxel at location r, the water and fat components share the same motion‐induced phase $\phi_{n,l}\left(r\right)$ in each shot l and for each chemical shift‐encoding step *n*. Solving jointly for the motion‐induced phase term $e^{-i\phi_{n,l}\left(r\right)}$ and the underlying water/fat components is a nonlinear optimization problem. Similar to water/fat‐separation approaches,^32^, ^36^ the iterative Gauss‐Newton method can be used to solve such a nonlinear problem. The motion‐induced phase term can be approximated as $e^{-i\left(\phi_{n,l}\left(r\right)+∆\phi_{n,l}\left(r\right)\right)}\approx e^{-i\phi_{n,l}\left(r\right)}\left(1-i∆\phi_{n,l}\left(r\right)\right)$ using first‐order Taylor expansion and can be updated in the next iteration of the Gauss‐Newton scheme. Substituting water/fat components by $\rho_{w}\left(r\right)=\rho_{w}\left(r\right)+\Delta \rho_{w}\left(r\right)$, $\rho_{f}\left(r\right)=\rho_{f}\left(r\right)+\Delta \rho_{f}\left(r\right)$ and neglecting second and higher order terms, Equation (1) can be written as:

(5) $$s_{n,l,j}\left(t\right)=\int \left[c_{j},\left(r\right),\;,\left(\rho_{w}\left(r\right)+\Delta \rho_{w}\left(r\right)\right),+,\sum_{m=1}^{M},\alpha_{m},c_{j},\left(r\right),\left(\rho_{f}\left(r\right)+\Delta \rho_{f}\left(r\right)\right),e^{-i2\pi \psi_{f,m}\left(\Delta {\mathrm{TE}}_{n}+t\right)}\right]e^{-i2\pi \psi_{B}\left(r\right)\Delta {\mathrm{TE}}_{n}}e^{-i\phi_{n,l}\left(r\right)}\left(1-i∆\phi_{n,l}\left(r\right)\right)e^{-ik_{t}\cdot r}\mathrm{dr}\;.\;$$

The unknown vector $∆Y={\left[∆{Ρ}_{w},∆{Ρ}_{f},∆\Phi_{1,1},\ldots ,∆\Phi_{N,1},\ldots ,∆\Phi_{1,L},\ldots ,∆\Phi_{N,L}\right]}^{T}$ can be formed and determined by minimizing

(6) $$\begin{array}{c} {\left\{∆{Ρ}_{w},∆{Ρ}_{f},∆\Phi_{1,1},\ldots ,∆\Phi_{N,1},\ldots ,∆\Phi_{1,L},\ldots ,∆\Phi_{N,L}\right\}}^{*}=\underset{\begin{array}{c} ∆\Phi_{n,l}\in {\mathbb{R}}^{Q}\\ \Delta {Ρ}_{w},\Delta {Ρ}_{f}\in {\mathbb{C}}^{Q} \end{array}}{\text{argmin}}{\left\|\hat{B}∆Y-∆S\right\|}_{2}^{2}, \end{array}$$

where $∆{Ρ}_{w}={\left[{{\Delta \rho }_{w}}^{1},\ldots ,\Delta {\rho_{w}}^{Q}\right]}^{T}$, $∆{Ρ}_{f}={\left[{{\Delta \rho }_{f}}^{1},\ldots ,\Delta {\rho_{f}}^{Q}\right]}^{T}$, $∆\Phi_{n,l}={\left[∆{\phi_{n,l}}^{1},\ldots ,∆{\phi_{n,l}}^{Q}\right]}^{T}$ for n=1,…,N;l=1,…,L, and $∆S=S-\hat{A}\bar{X}$ with the current estimated $\bar{X}$ from the last iteration, with the coefficient matrix $\hat{B}$ of the Gauss‐Newton error system, which will be given below. To enforce smoothness of the estimated phase maps, a 2D triangular window^11^ is applied in k‐space for each iteration. By combining all shots into a large system matrix, while estimating only one magnitude for water and fat, the resulting problem is better conditioned compared with separating the water/fat of each shot individually. However, the phase is allowed to differ between shots reflecting the physiological motion effects. The total signal model of Equation 3 can be rewritten in terms of ∆Y as:

(7) $$\begin{array}{c} \begin{array}{c} S=\hat{A}X+\hat{B}∆Y. \end{array} \end{array}$$

The matrix $\hat{B}$ can be calculated as

(8) $$\hat{B}∆Y=\hat{A}\left[\left(∆{Ρ}_{w}-i{Ρ}_{w}∆\Phi_{1,1}\right),\ldots ,\left(∆{Ρ}_{w}-i{Ρ}_{w}∆\Phi_{N,1}\right),\ldots ,\left(∆{Ρ}_{w}-i{Ρ}_{w}∆\Phi_{1,L}\right),\ldots ,\left(∆{Ρ}_{w}-i{Ρ}_{w}∆\Phi_{N,L}\right)\right.,\;\begin{array}{c} \left.\left(∆{Ρ}_{f}+i{Ρ}_{f}∆\Phi_{1,1}\right),\ldots ,\left(∆{Ρ}_{f}-i{Ρ}_{f}∆\Phi_{N,1}\right),\ldots ,\left(∆{Ρ}_{f}-i{Ρ}_{f}∆\Phi_{1,L}\right),\ldots ,\left(∆{Ρ}_{f}-i{Ρ}_{f}∆\Phi_{N,L}\right)\right], \end{array}\;$$

where ${Ρ}_{w}/{Ρ}_{f}$ are the water/fat images calculated from the last Gauss‐Newton iteration. $\hat{A}$ is the matrix system calculated through Equation 3, in which the diffusion phase operator $\hat{\Phi }$, containing all the shot‐to‐shot phase terms $\Phi_{n,l}={\left[{\phi_{n,l}}^{1},\ldots ,{\phi_{n,l}}^{Q}\right]}^{T}$ for n=1,…,N;l=1,…,L and all voxels Q, also estimated from the last Gauss‐Newton iteration.

Joint water/fat separation with motion‐induced phase map estimation can therefore be summarized as follows for each b‐value and diffusion direction:

1. Initialize phase maps $\Phi_{n,l}$ (see section 2.4).
2. Estimate water and fat images ${Ρ}_{w}$, ${Ρ}_{f}$ by solving Equation 4 with the current phase maps $\Phi_{n,l}$.
3. Calculate the error system matrix $\hat{B}$ (Jacobian matrix) using the current ${Ρ}_{w}$, ${Ρ}_{f}$, and $\Phi_{n,l}$ estimates via Equation 8.
4. Calculate the updated error of the phase map, $∆\Phi_{n,l}$, and update the error with $\Phi_{n,l}=\Phi_{n,l}+∆\Phi_{n,l}$ for each chemical shift‐encoding step n and shot l using Equation 6.
5. Enforce the smoothness of each phase term $e^{{\mathrm{i\Phi}}_{n,l}}$ applying a triangular window in k‐space.
6. Repeat the preceding steps 2–5 until the normalized residual norm of the Gauss‐Newton loop drops below a threshold or a maximum number of iterations is reached.

### 2.4 Initialization for the shot‐to‐shot phase maps

One of the challenges for combining self‐navigation and water/fat separation is how to avoid that the estimation is not trapped into a local minimum. This is also one major difficulty for other water/fat separation algorithms, when dealing with the exponential phase terms of fat and B_0_ off‐resonances.^41^, ^42^ In solving Equation 1, even when an accurate B_0_ map is provided, the needed estimation of each shot‐specific, motion‐induced phase term may cause inaccurate water/fat estimations. To avoid inaccurate convergence of the algorithm, MUSE^15^ can be a good candidate for initializing the phase maps. However, in EPI images without fat suppression, the spatial displacement of fat signals should also be addressed for proper phase extraction. One solution is to use a sensitivity encoding for fast MRI (SENSE)‐based water/fat separation^43^, ^44^, ^45^ instead of conventional SENSE in the MUSE implementation. This MUSE‐like water/fat‐resolved algorithm will be referred to as “water‐fat MUSE”. Water/fat components can be disentangled by solving the SENSE‐based water/fat separation^43^, ^44^, ^45^ using a similar system as in Equation 3), dropping B_0_ ( ${\hat{\Psi }}_{B}$) and diffusion phase ( $\hat{\Phi }$) operators and calculating water/fat images for each chemical shift‐encoding point n and each shot l. Solving for N chemical shift‐encoding points and L shots data simultaneously, the joint system can be constructed as:

(9) $$\begin{array}{c} S=\left[\;\hat{I}\;\hat{I}\right]\left[\;\begin{array}{c} \hat{F}\\ 0 \end{array}\;\begin{array}{c} 0\\ {\hat{\Psi }}_{f}\hat{F} \end{array}\;\right]\left[\;\begin{array}{c} {\hat{C}}_{l}\\ 0 \end{array}\;\begin{array}{c} 0\\ {\hat{C}}_{l} \end{array}\;\right]\left[\;\begin{array}{c} {\tilde{Ρ}}_{w}\\ {\tilde{Ρ}}_{f} \end{array}\right]={\hat{A}}_{l}\tilde{X}, \end{array}$$

where the coil sensitivity operator ${\hat{C}}_{l}$ is slightly modified to disable the shot combination step (i.e., every shot data will be treated as an independent undersampled case), $\tilde{X}={\left[{\tilde{Ρ}}_{w},{\tilde{Ρ}}_{f}\right]}^{T}={\left[{\tilde{Ρ}}_{w,1,1},\ldots ,{\tilde{Ρ}}_{w,N,1},\ldots ,{\tilde{Ρ}}_{w,1,L},\ldots ,{\tilde{Ρ}}_{w,N,L,}{\tilde{Ρ}}_{f,1,1},\ldots ,{\tilde{Ρ}}_{f,N,1},\ldots ,{\tilde{Ρ}}_{f,1,L}\ldots ,{\tilde{Ρ}}_{f,N,L}\right]}^{T}$ contains all individual water/fat estimations ${\tilde{Ρ}}_{w/f,n,l}$ for n=1,…,N;l=1,…,L, and ${\hat{A}}_{l}$ is the corresponding system matrix. Like MUSE, a total‐variation (TV) regularization can be used to enforce the smoothness of the water/fat images. Equation 9 can be solved and gives a decent initial guess for the phase and thus for water and fat to start the full MSND iteration, as:

(10) $$\begin{array}{c} {\left\{{\tilde{Ρ}}_{w},{\tilde{Ρ}}_{f}\right\}}^{*}=\underset{{\tilde{Ρ}}_{w},{\tilde{Ρ}}_{f}\in {\mathbb{C}}^{Q}}{\text{argmin}}{\left\|{\hat{A}}_{l}\tilde{X}-S\right\|}_{2}^{2}+\mathrm{\lambda TV}\left(\tilde{X}\right), \end{array}$$

where λ is the regularization factor and TV is the total‐variation operator^32^, ^46^ on each ${\tilde{Ρ}}_{w,n,l}$ and ${\tilde{Ρ}}_{f,n,l}$, separately. Next, to extract only the pure motion‐induced shot‐to‐shot phase errors, the same approach as proposed by Moeller et al.^17^ can be adopted. For a given b‐value, for each chemical shift‐encoding point n and shot l, a merged phase map ${\tilde{\Phi }}_{n,l}$ can be calculated by a simple weighted summation of ${\tilde{Ρ}}_{w,n,l}+{\tilde{Ρ}}_{f,n,l}$ followed by the phase extraction. This water‐fat MUSE can also be applied to the b = 0 s/mm^2^ data once to calculate phase maps ${\tilde{\Phi }}_{n,l}^{b_{0}}$, which contain the same base phase information apart from the diffusion phases in ${\tilde{\Phi }}_{n,l}^{b_{i}}$ ( $b_{i}\;$> 0 s/mm^2^). The pure diffusion phase can be calculated by the subtraction ${\tilde{\Phi }}_{n,l}^{\text{init},\;b_{i}}={\tilde{\Phi }}_{n,l}^{b_{i}}-{\tilde{\Phi }}_{n,l}^{b_{0}}$, where ${\tilde{\Phi }}_{n,l}^{\text{init},\;b_{i}}$ is the phase map used for initialization of $b_{i}$ data. Then ${\tilde{\Phi }}_{n,l}^{\text{init},\;b_{i}}$ can be used as a good starting point for the phase‐estimation step described in the last subsection.

### 2.5 Coil‐sensitivity maps calibration and B_0_ effect correction

Like in all SENSE‐based methods,^11^, ^14^, ^15^, ^16^, ^17^, ^47^ it is crucial that the geometric distortions of the CSM data, often acquired via a prescan, and the EPI data, match sufficiently well. For instance, this can be achieved by demodulating the EPI data in k‐space using a B_0_ map,^4^, ^48^ when it is known. However, this can be impractical because of the computational burden imposed by the repeated Fourier transforms with B_0_ modulation.^49^, ^50^ Instead, calibrating CSMs to match the EPI conditions in a distorted manner only once can be a more time‐efficient approach.

When the B_0_ map is not known as a prior, one solution is to estimate the CSM and B_0_ map from the EPI data itself, preferably from the non‐DW (b = 0 s/mm^2^) data.^11^, ^15^ This autocalibration can be achieved in a two‐step manner. First, a B_0_ map estimation can be performed via published methods,^32^, ^50^, ^51^ which take the spatial displacements of fat into account. Second, the calibration can be accomplished using Eigenvector‐based iTerative Self‐consistent Parallel Imaging Reconstruction (ESPIRiT),^52^ estimating merged water‐fat (position‐corrected) CSMs. Before applying ESPIRiT, it is necessary to perform a water/fat separation step for each coil data based on the known B_0_ map to eliminate the spatial displacement of fat. As an alternative, established water‐fat J‐SENSE^43^, ^44^ approaches can be used that correct automatically for the chemical shift effect of fat in the algorithm.

Alternatively, the B_0_ map and CSM can also be jointly estimated from the b = 0 s/mm^2^ data by using the Gauss‐Newton method to form a one‐step only autocalibration. This can be achieved by reformulating Equation 5 with another error term $c_{j}\left(r\right)=c_{j}\left(r\right)+\Delta c_{j}\left(r\right)$ as described in established methods,^43^, ^44^, ^53^ in which $\Delta c_{j}\left(r\right)$ is the error of CSM updated to the next Gauss‐Newton iteration. Because of the absence of diffusion‐sensitizing gradients in the b = 0 s/mm^2^ data, the phase terms $e^{-i2\pi \psi_{B}\left(r\right)\Delta {\mathrm{TE}}_{n}}e^{-i\phi_{n,l}\left(r\right)}$ for each shot l and chemical shift‐encoding point n in Equation 1) are only contributed from B_0_ inhomogeneity $\psi_{B}\left(r\right)$ (i.e., $\phi_{n,l}\left(r\right)=0$). Similarly, as described in Equations (5) and 6, the water/fat components $\rho_{w}\left(r\right)$ and $\rho_{f}\left(r\right)$, B_0_ phase term $\psi_{B}\left(r\right)$, and CSM $c_{j}\left(r\right)$ for each coil j can be jointly estimated. As a common challenge for the B_0_ map estimation, further constraints may be used to avoid being trapped by local minima, as described in the establishing works.^32^, ^41^, ^42^, ^54^ Likewise, smoothness of CSM can also be enforced with the published approaches.^40^, ^53^, ^55^, ^56^ Notably, in this work, it is intended to differentiate the B_0_ map from the motion‐induced phase terms to further correct the geometric distortion of the DW images as a postprocessing step.^30^, ^32^, ^48^, ^49^

## 3 METHODS AND MATERIALS

### 3.1 MRI acquisition

Experiments were conducted with 14 healthy subjects using a 3‐T scanner (Philips, Best, The Netherlands) with informed consent obtained and approved by the local ethics committee. The sequence parameters can be found in Table 1. All scans in the leg/head–neck region were acquired with an eight‐channel knee coil or 16‐channel head–neck coil, respectively. Coil‐sensitivity mapping was performed using standard Philips gradient‐echo prescan procedures, with a voxel size of 11 × 11 × 11 mm^3^, and TR/TE = 4.2/0.57 ms for all scans. Three‐point chemical shift ( ΔTE = 0.2/1.0/1.8 ms) encoded spin‐echo DW ms‐EPI data were acquired to sample the in‐phase period (∼2.3 ms at 3 T) between water and methylene fat (−3.4 ppm) almost symmetrically.^32^ For each measurement, four slices with a gap of 10 mm and three b‐values (b = 0, 300, and 600 s/mm^2^) were measured. Only a single diffusion direction was applied to test the reconstruction algorithm. In all scans, for each shot an extra 2D navigator^7^ was acquired for comparison (see Figure 1A). For one head–neck experiment, one volunteer was additionally measured with fat suppression by SPIR (no chemical shift encoding) and a Philips gradient‐echo mDixon protocol for comparison. It should be acknowledged that two datasets from the original study of the IDE algorithm in Dong et al.^32^ are included in this work for comparison purposes (one lower leg dataset, 6‐shot; and one shoulder dataset, 6‐shot, included in the apparent diffusion coefficient [ADC] analysis in the supporting information (S.2)).

**TABLE 1:** Sequence parameters

Sequence name	Anatomy	Resolution (mm^3^)	Matrix size	Number of shots	TE/TE_nav_ */TR (ms)	Echo spacing (ms)	Shot duration (ms) with/without 2D‐navigator
Chemical shift‐encoded DW ms‐EPI	leg	1.5 x 1.5 x 4	160 × 150	6	62/98/2000	0.786	73/102
Chemical shift‐encoded DW ms‐EPI	leg	1.2 x 1.2 x 4	168 × 162	6	64/117/2000	1.258	83/122
Chemical shift‐encoded DW ms‐EPI	leg	1.5 x 1.5 x 4	152 × 148	4	69/113/2000	0.746	85/117
Chemical shift‐encoded DW ms‐EPI	head–neck	1.5 x 1.5 x 4	160 × 150	6	62/98/2000	0.786	73/102
Chemical shift‐encoded DW ms‐EPI	head–neck	1.5 x 1.5 x 4	152 × 148	4	69/113/2000	0.746	85/117
Chemical shift‐encoded DW ms‐EPI	head–neck	2.0 x 2.0 x 4	116 × 102	2	72/120/2000	0.633	90/127
DW ms‐EPI (SPAIR on)	leg	1.5 x 1.5 x 4	160 × 150	6	62/98/2000	0.786	73/102
DW ms‐EPI (SPIR on)	head–neck	1.5 x 1.5 x 4	160 × 150	6	62/98/2000	0.786	73/102
DW ms‐EPI (SPIR on)	head–neck	1.5 x 1.5 x 4	152 × 148	4	69/113/2000	0.746	35/24
FFE mDixon	head–neck	1.5 x 1.5 x 4	152 × 148	‐	3.6/−/32	‐	7

Abbreviations: DW, diffusion‐weighted; FFE, fast field echo (gradient echo); ms‐EPI, multishot echo‐planar imaging; SPAIR, SPectral Attenuated Inversion Recovery; SPIR, Spectral Presaturation with Inversion Recovery.

* TE_nav_: the TE of the navigator echo.

### 3.2 Reconstruction

The proposed MSND reconstruction method was implemented in Python 3.7 using a PC with an Intel Core i7 CPU (3.0 GHz, eight cores) with 64 GB of RAM, and an NVIDIA GeForce RTX 2080 Ti GPU. A schematic reconstruction flowchart is shown in Figure 1B. The two‐step CSM and B_0_ map estimated from the b = 0 s/mm^2^ data were used to reconstruct the diffusion b > 0 s/mm^2^ data. Unless stated otherwise, the CSM was autocalibrated using the ESPIRiT‐based approach (implementation in the “SigPy” toolbox^57^ was adopted). The B_0_ map as well as water and fat masks were only calculated once using the IDE^32^ algorithm. The water/fat masks were multiplied with the coil sensitivity‐weighting operator $\hat{C}$ for the water/fat channels to stabilize the following reconstruction steps. This is also a common constraint used for routine SENSE^40^ reconstruction and SENSE‐based chemical species separation.^43^, ^44^ The thresholds for water/fat masks were empirically set to 0.03 of the maximum amplitude of the water/fat images signal, respectively. Before masking the CSM, binary erosion (one iteration) was performed on each mask to remove noisy pixels outside the subject, with a subsequent binary expansion step (three iterations) to prevent any potential edge effects.

For the self‐navigation step, in each iteration of the Gauss‐Newton loop, the two least‐square systems in Equations 4 and 6 were solved with conjugate gradient (CG). Convergence was assumed when the normalized residual norm (tolerance) dropped below 10^−3^ or the maximum number of CG iterations exceeded 20. The initializations for the phase maps using water‐fat MUSE were also calculated through CG, with a tolerance of 10^−2^ and maximal iterations of 10 of each least square system. The regularization factor λ of the total variation was set to 0.01. For the Gauss‐Newton loop, the tolerance value as a stopping criterion was set to 10^−4^. In most cases, the maximum number of iterations was less than or equal to eight to reach the tolerance. All the above reconstruction parameters were tested in all datasets and were chosen empirically. The 2D triangular window^11^ widths were set empirically to 5/7 and 1/2 of the matrix size for the 1.5/1.2 mm in‐plane resolution measurements, respectively, to enforce smoothness. To speed up the computation, all the head–neck data acquired with the 16‐channel coil were compressed into eight channels through a standard coil compression algorithm.^58^ All reconstructions were performed using a 6‐peak fat model,^32^ in which the relative weights are self‐calibrated with respect to a TE of about 60 ms.

First, an experiment was conducted comparing the reconstruction results (1) using an unmodified “prescan CSM”, (2) using a CSM acquired with the prescan but distorted by a “B_0_‐based calibration”, and (3) using ESPIRiT (two‐step autocalibration). This is to better illustrate the importance of eliminating the mismatch caused by geometric distortions between the CSM obtained from a prescan and EPI images. The B_0_‐based calibration was achieved by using a B_0_ map to distort the CSM acquired with the conventional gradient echo‐based prescan to spatially match the geometric distortions in the EPI data. This can be accomplished by performing an “inverted” geometric distortion correction based on the already estimated B_0_ map^4^, ^30^ (acquired from IDE at b = 0 s/mm^2^) for each CSM, using inverse conjugate phase reconstruction (CPR).^46^, ^59^ Furthermore, the reconstruction result using the “one‐step” autocalibration is shown in the supporting information (S.3) for further comparison.

For the remainder of the experiments, the two‐step CSM autocalibration frame was used to estimate both the B_0_ map and CSM. Water‐fat MUSE‐based initialization was performed for all head–neck data. For leg data, the initialization of phase maps was set to zero. One water/fat separation reference approach is the IDE^32^ algorithm, which utilizes measured extra 2D navigators (Figure 1A) to correct for shot‐to‐shot phase variations. All the datasets reconstructed with the IDE algorithm were using the reconstruction parameter settings described in the original paper.^32^ To show the effects of extra or self‐navigation, the comparison was conducted in the leg and head–neck measurements. In addition, ADC fittings were performed for both IDE and MSND algorithms in the head–neck data. Furthermore, the estimated B_0_ map was used for a final geometric distortion correction using CPR^46^, ^59^ in a postprocessing step.

### 3.3 Evaluation criteria

The coefficient of variation ( CoV) was employed as a quantitative measure of the image quality. This is a measure of signal intensity spread defined as CoV=SD/mean, where SD and mean are the standard deviation and mean of the signal intensity calculated within each region of interest (ROI). The evaluation was conducted on four leg datasets (6‐shot, b = 600 s/mm^2^). The ROIs were drawn manually on the water images for five muscles in the knee scans and for four muscles in the calf scans: vastus lateralis muscle, vastus medialis muscle, semimembranosus muscle, biceps femoris, and sartorius muscle for the knee; and medial head of gastrocnemius, soleus, tibialis posterior, and lateral head of gastrocnemius for the calf. The assessment was performed for each of the four slices per volunteer, resulting in 20 CoVs for the knee, and 16 CoVs for the calf scans. A comparison of CoVs between MSND and IDE was performed using the paired *t*‐test for all calculated CoVs and a *p*‐value of less than 0.05 was considered statistically significant. To further confirm the accuracy/improvement of the proposed approach, ADC fittings were performed for both MSND and fat saturation techniques in both B_0_ homogeneous leg and B_0_ inhomogeneous head–neck regions and are illustrated in the supporting information (S.2).

## 4 RESULTS

**FIGURE 2:**
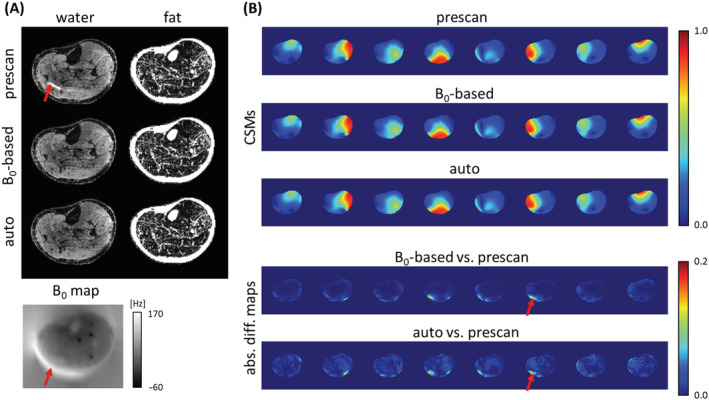
Impact of coil‐sensitivity maps (CSMs) with/without calibration. (A) Diffusion‐weighted water/fat images (6‐shot, in‐plane resolution = 1.5 mm, b = 600 s/mm^2^) estimated via Model‐based Self‐Navigated water/fat Decomposition using CSMs derived from prescan data, from B_0_‐based calibrated prescan data, and from autocalibrated (two‐step) multishot echo‐planar imaging (EPI) data itself. Some artefacts can be seen in the water image, reconstructed with the original prescan CSM. (B) The corresponding CSMs of the three methods are shown along with the absolute difference maps with respect to the prescan. Differences, partly marked by red arrows, are mainly caused by a mismatch between the CSMs and the EPI images due to B_0_‐induced geometric distortion (see the associated B_0_ map displayed in (A)). Such artifacts can be avoided by using either auto‐ or B_0_‐based calibration methods

Figure 2A shows a comparison of the MSND water/fat separation results using CSM information obtained with three different approaches, as shown in Figure 2B. Because of the relatively large local B_0_ inhomogeneity, the EPI images are distorted, leading to the geometrical mismatch of the CSMs obtained from a conventional gradient echo prescan. Such misalignment compromises the data consistency in the MSND, resulting in artefacts in the water images. These artefacts were reduced by either using a B_0_ map to adapt prescan CSMs to the EPI scanning conditions, or autocalibrating the CSM from the EPI data itself using ESPIRiT. The B_0_ map used in this comparison was estimated using IDE using nondiffusion‐sensitized (b = 0 s/mm^2^) data.

**FIGURE 3:**
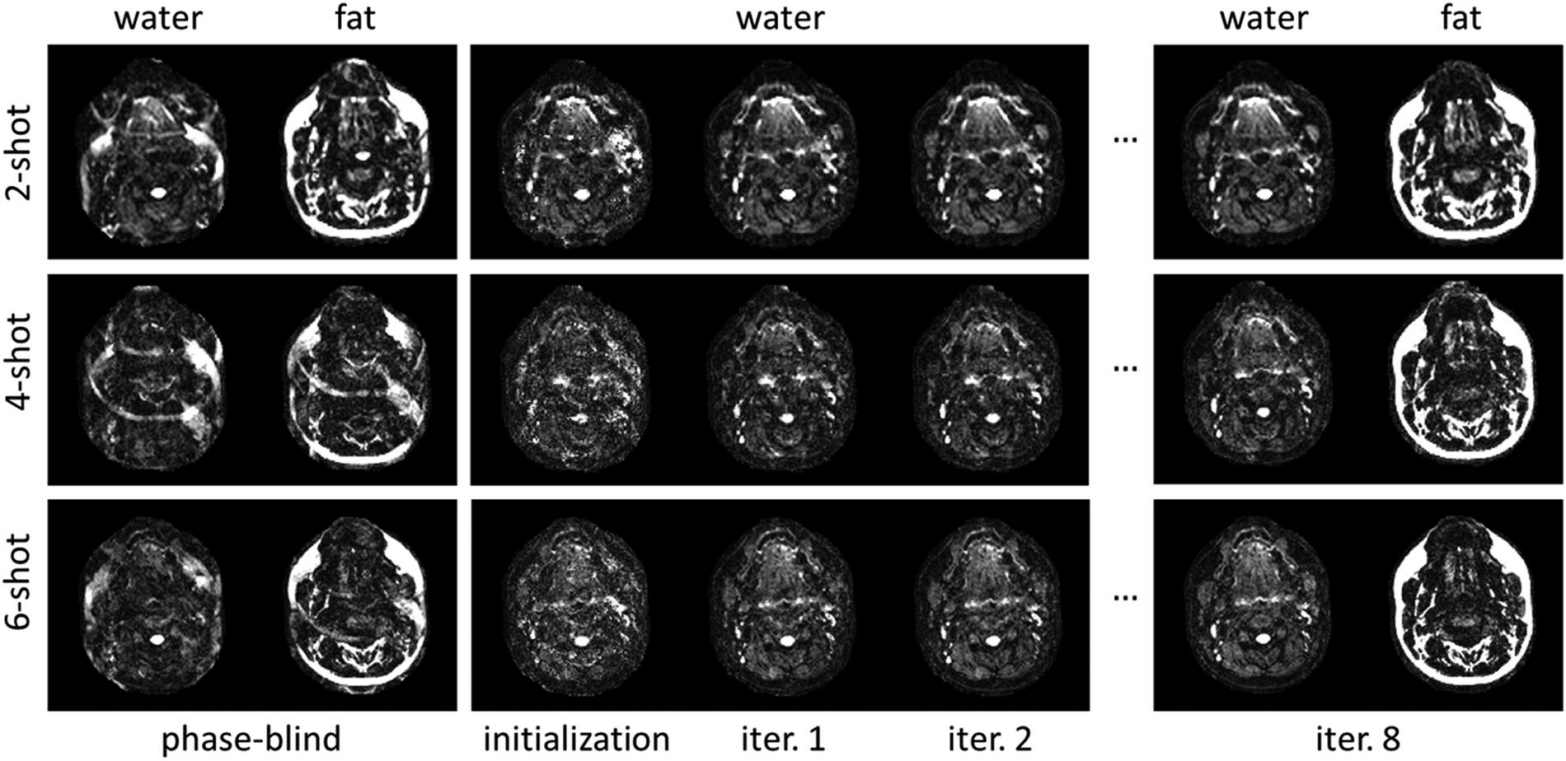
Reconstruction to illustrate convergence of Model‐based Self‐Navigated water/fat Decomposition (MSND). Three head–neck diffusion‐weighted (DW) datasets (b = 600 s/mm^2^) with different numbers of shots of the same slice are shown. (Left) Water and fat images reconstructed using water‐fat separation ignoring the shot‐to‐shot phase errors (zero‐valued phase maps were used). (Middle) Water images reconstructed with water‐fat multiplexed sensitivity‐encoding (MUSE), used as initialization for MSND phase maps, and water images after the first two iterations of MSND. (Right) The water/fat images obtained with MSND after the eighth iteration. With the water‐fat MUSE initialization, the MSND algorithm can separate the fat from the water images, while correcting shot‐to‐shot phase errors for the multishot echo‐planar imaging data, even for large segmentation factors (6‐shot)

Figure 3 shows examples of the iterative evolution of DW images reconstructed with MSND compared with the water/fat separation using the same model but ignoring shot‐to‐shot phase errors. The three datasets were measured in the same slice but with a different number of shots and on different resolutions (see Table 1). The reconstructed results with zero‐valued phase maps clearly show the impact of the motion‐induced shot‐to‐shot phase variations. In this case, the fat signals cannot be correctly removed from the water images because the shot‐to‐shot phase errors disrupt the phase correlations between data acquired at different ΔTE. After initializing with water‐fat MUSE and running MSND, the fat signals were successfully separated from the DW water images on all three datasets, while also correcting the shot‐specific phase variations. In addition, the impact of using or not using water‐fat MUSE as initialization can be found in the supporting information (S.4). All three reconstructions were stopped at the eighth iteration for comparison.

**FIGURE 4:**
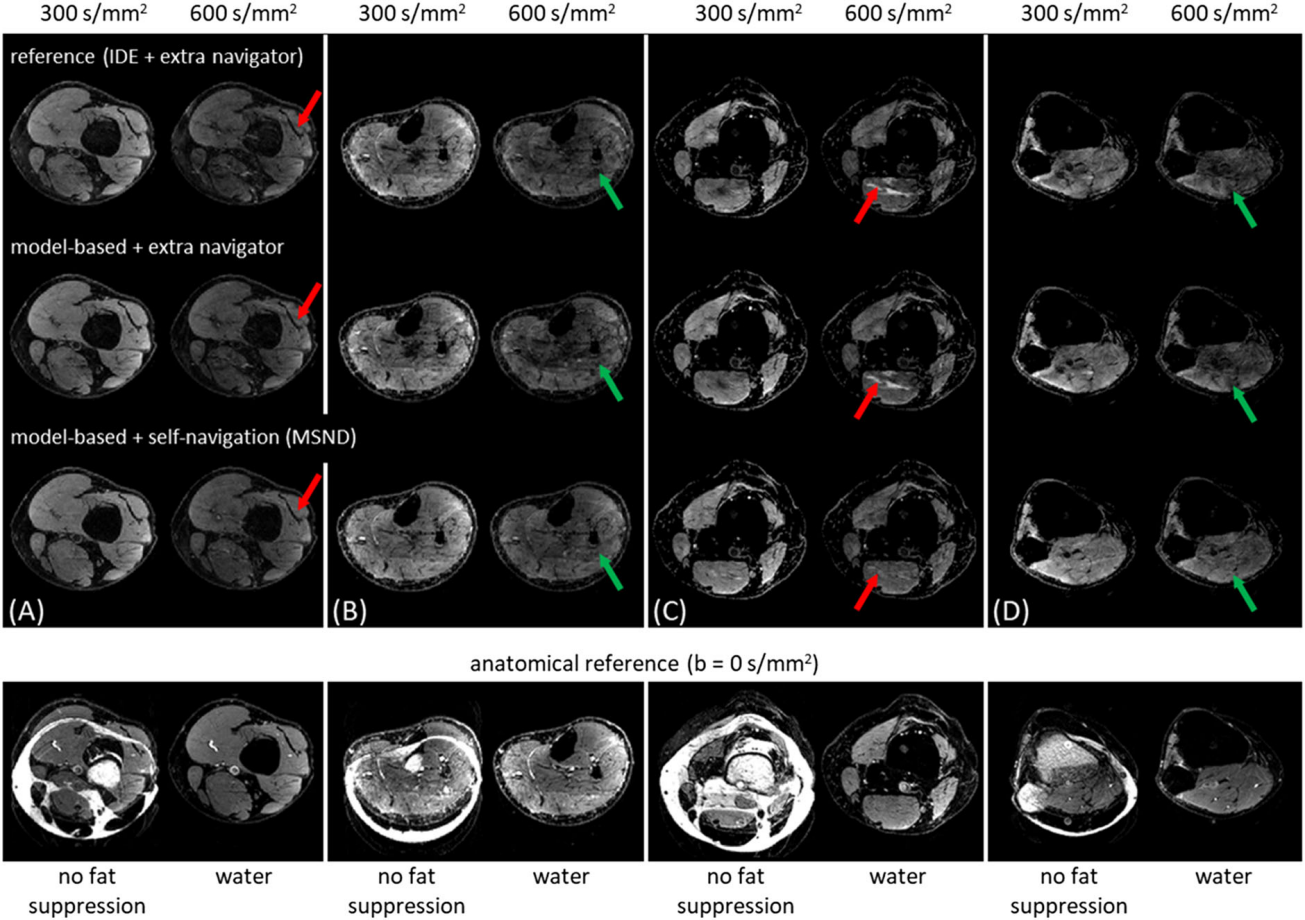
Comparison among three water/fat separation methods in four volunteers’ leg data (6‐shot). The image‐based, water/fat decomposition approach for echo‐planar imaging (IDE) and the model‐based approach with extra navigator show some artefacts in the final water images, mainly due to the unsuppressed fat signals (red arrows in volunteers 1 (A) and 3 (C)) and lower signal‐to‐noise ratio (green arrows in volunteers 2 (B) and 4 (D)) of the navigator. These can be mitigated through Model‐based, Self‐Navigated water/fat Decomposition (MSND) using self‐navigation (marked in red arrows). In the bottom row, the b = 0 s/mm^2^ image of the first chemical shift‐encoding step using SENSE‐based shot‐combination (no fat suppression), and the associated water image reconstructed using the model‐based, water‐fat separation are shown as the anatomical references

Figure 4 compares three reconstruction methods for data measured in four different volunteers: IDE with measured extra navigator, the proposed extended model‐based water/fat separation approach with measured extra navigator (no self‐navigated phase estimation involved), and the same model with self‐navigation (MSND). The second reconstruction method was introduced to distinguish between the impact of the model‐based solution and self‐navigation. The anatomical reference data (b = 0 s/mm^2^) are shown as well. The images without fat suppression were reconstructed using a simple SENSE‐based shot‐combination^7^ of the data for one given (first) chemical shift‐encoding step. The associated water image (b = 0 s/mm^2^) was reconstructed by performing the proposed model‐based water/fat separation without the self‐navigation step using the B_0_ map estimated from IDE. The artefacts present in the DW water images of the first two methods can be reduced by using self‐navigation.

**FIGURE 5:**
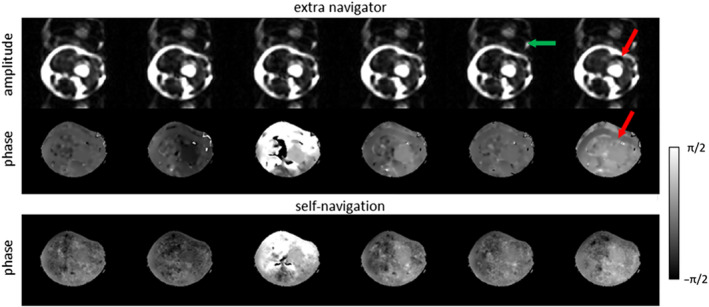
Comparison of extra navigators (low resolution) and self‐navigated phase maps for 6‐shot, diffusion‐weighted, multishot echo‐planar imaging data (volunteer 1 in Figure 4, b = 600 s/mm^2^). The navigator amplitude/phase and the phase maps estimated by self‐navigation at one given ΔTE are shown. The extra navigator was acquired at TE = 98 ms showing a relatively low water amplitude compared with the fat. The fat signals are shifted in the phase‐encoding direction and are overlapping with water signals (red arrows). Some fat ghosting can be seen in the extra navigator images (green arrow). These challenges can be resolved using Model‐based Self‐Navigated water/fat Decomposition by simultaneously correcting for fat off‐resonance effects and shot‐to‐shot phase variations

Figure 5 shows the estimated phase maps obtained with MSND for the first chemical shift‐encoding point (6‐shot) and the corresponding magnitude/phase of the extra navigator for comparison. It clearly shows that the artefacts marked in the data of the first volunteer in Figure 4A are mainly caused by the shifted subcutaneous fat signals overlapping with the water signal. This leads to an ambiguous measure of the phase information at these locations when the extra navigator is used.

**FIGURE 6:**
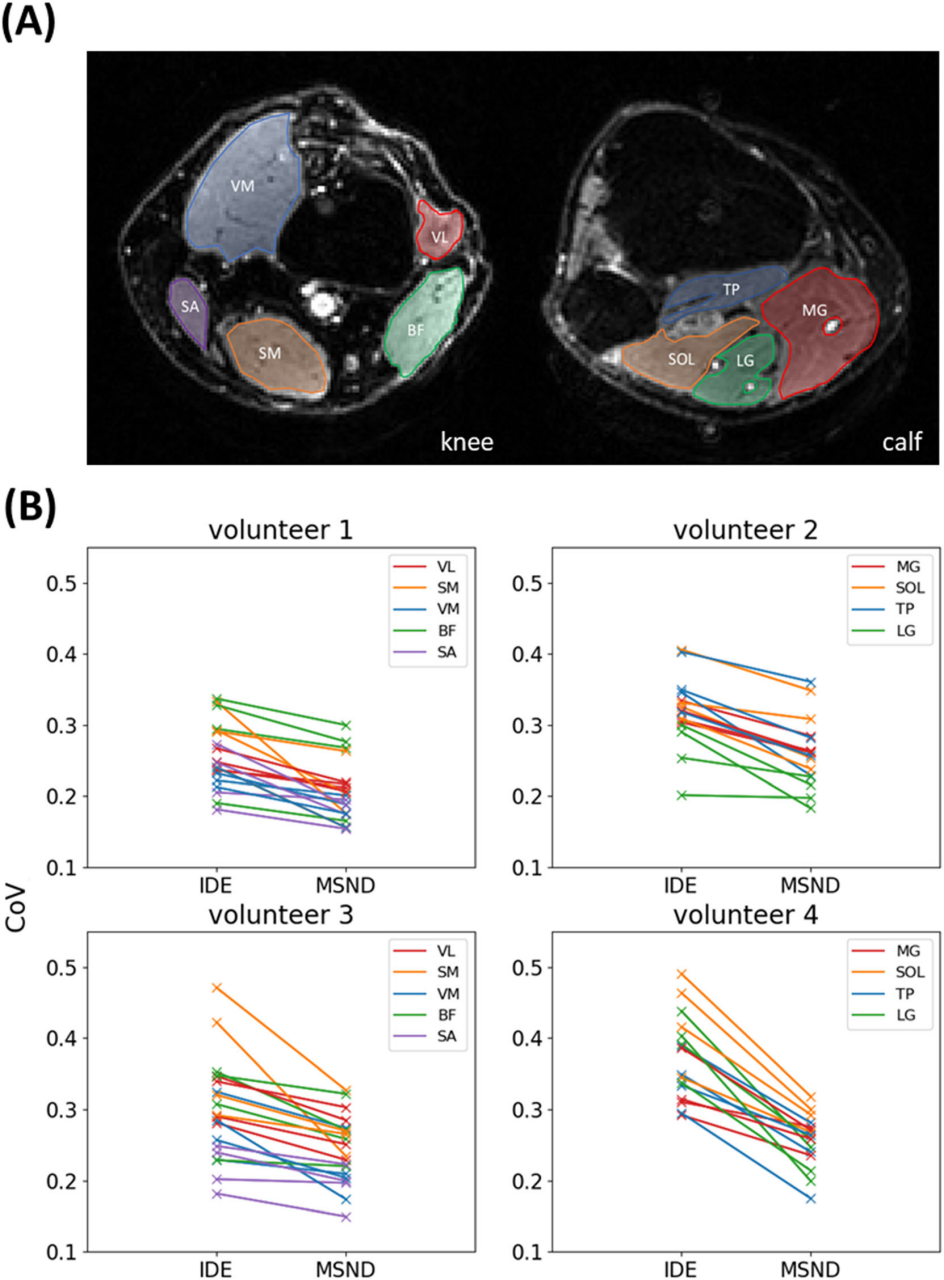
Quantitative comparison between image‐based, water/fat decomposition approach for echo‐planar imaging (IDE) and Model‐based, Self‐Navigated water/fat Decomposition (MSND) water images (b = 600 s/mm^2^). (A) An example of region of interest (ROI) selections for the knee and the calf, indicating different muscle groups for which the comparison is made. The coefficient of variation (CoV) was calculated for each ROI and each slice. (B) CoV for IDE and MSND. All slopes connecting IDE and MSND readings are negative (although may vary because of anatomical differences), showing improved performance by MSND. BF, biceps femoris; LG, lateral head of gastrocnemius; MG, medial head of gastrocnemius; SA, sartorius muscle; SM, semimembranosus muscle; SOL, soleus; TP, tibialis posterior; VL, vastus lateralis muscle; VM, vastus medialis muscle

Figure 6 shows a statistical comparison between IDE and MSND. For all volunteers, MSND results show lower CoV than the IDE results (statistically significant, *p* < 0.05). This can also be observed visually in Figure 4, where some of the artifacts shown in the IDE results are absent when using MSND.

**FIGURE 7:**
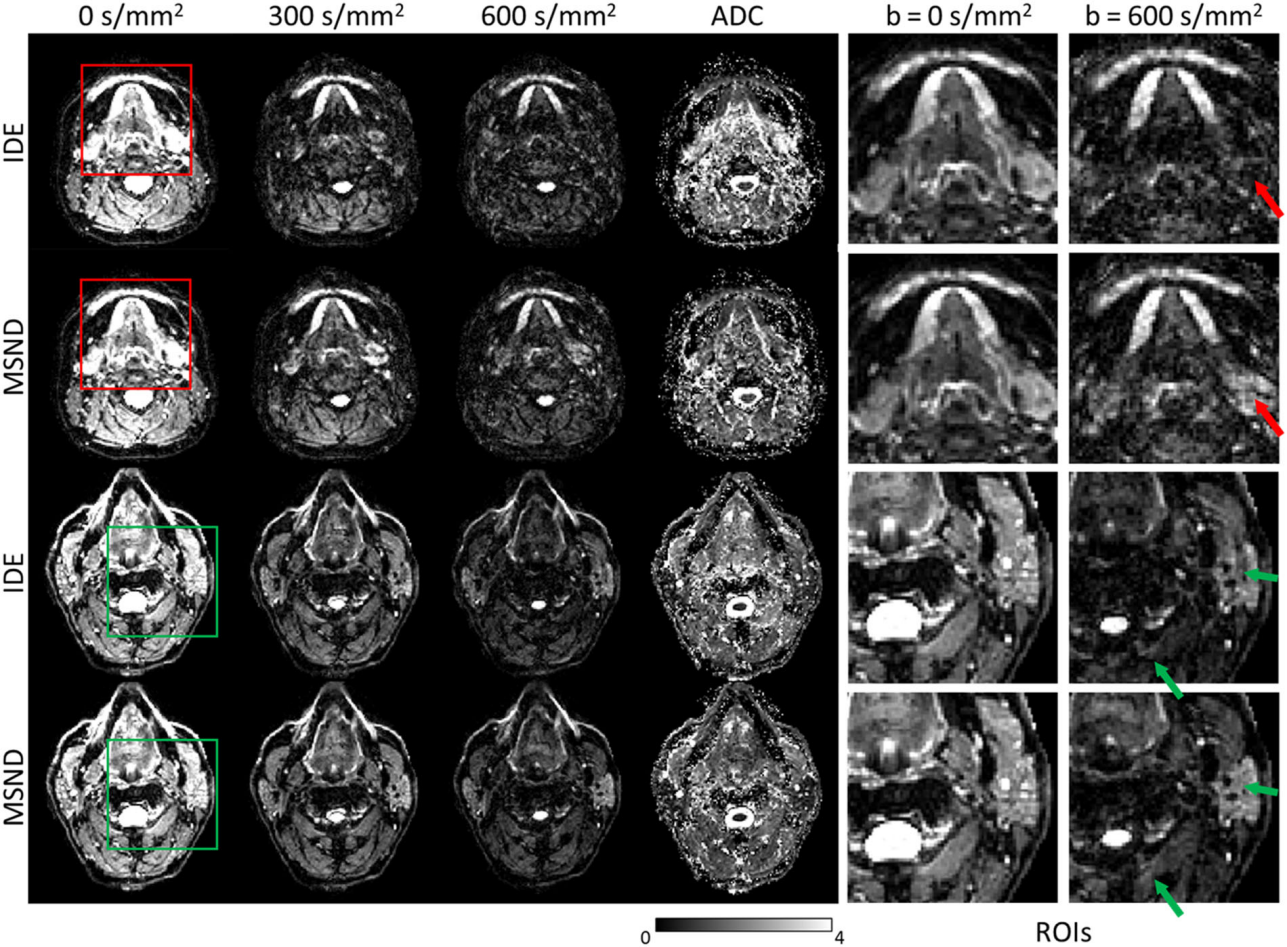
Comparison of the reconstructed image‐based, water/fat decomposition approach for echo‐planar imaging (IDE) and Model‐based Self‐Navigated water/fat Decomposition (MSND) water images in the head–neck region (two volunteers; 4‐shot, diffusion‐weighted [DW] multishot echo‐planar imaging data). Two regions of interest (ROIs) are selected for each volunteer in the b = 0 and b = 600 s/mm^2^ data and are displayed with zoom. There is no visible difference between b = 0 s/mm^2^ images reconstructed with the two methods. In the b = 600 s/mm^2^ case, signal loss/additional artefacts can be seen in the IDE‐reconstructed DW water images (red/green arrows). MSND can avoid such artefacts and produces improved quality water images. The consequence of this can be seen in the apparent diffusion coefficient (ADC) maps (10^−3^ mm^2^/s), where IDE shows abnormal ADC values in such artefact‐present regions. The top two rows and the bottom two rows belong to two separate volunteers

Figure 7 shows the performance of IDE and MSND in the head–neck region of two different volunteers. The results show that MSND produces improved water‐only images and better ADC maps compared with extra‐navigated results.

**FIGURE 8:**
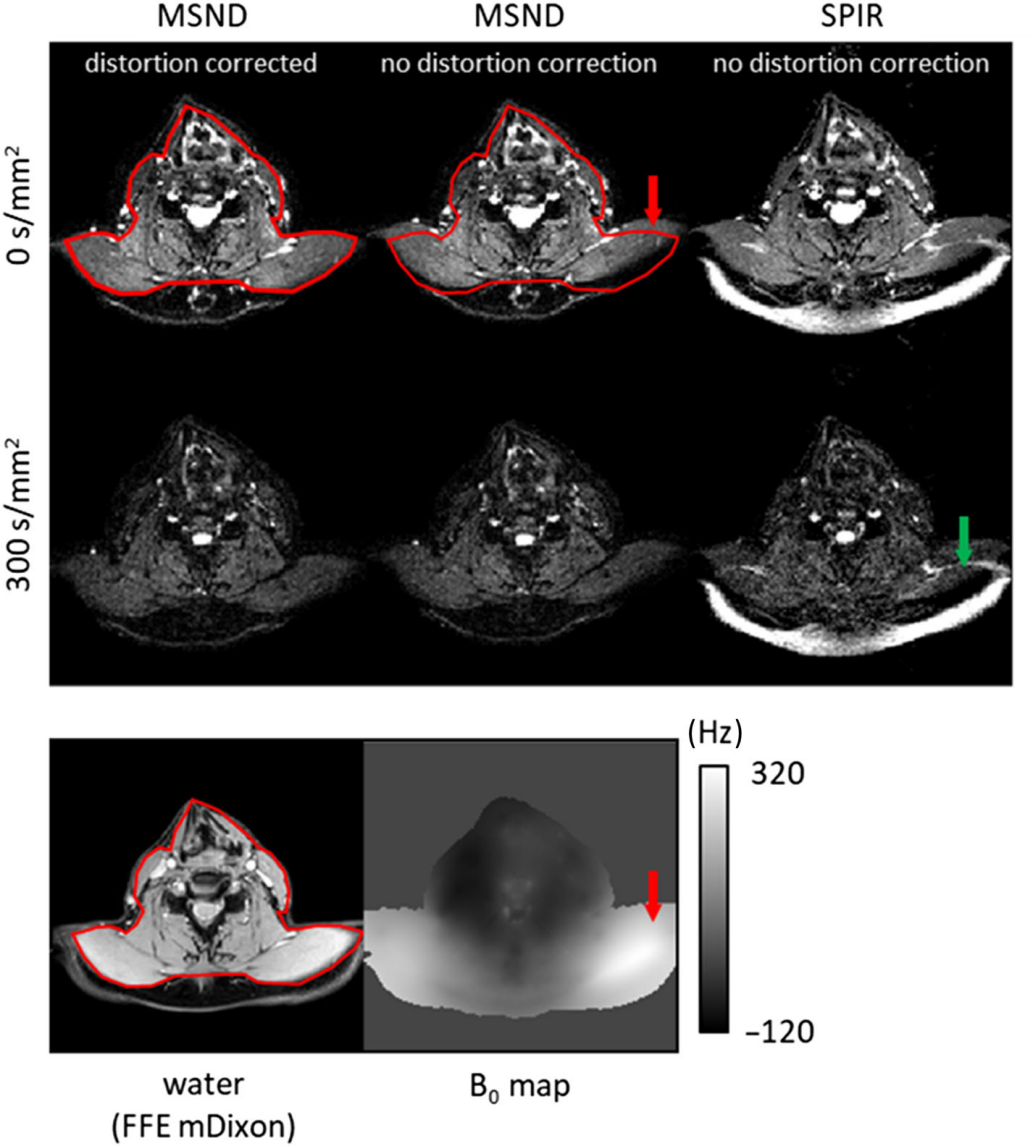
Effects of severe B_0_ inhomogeneities. The Model‐based Self‐Navigated water/fat Decomposition (MSND) water results of one subject's shoulder (4‐shot) at b = 0 and b = 300 s/mm^2^ with distortion correction as a postprocessing step are compared with MSND without distortion correction and with Spectral Presaturation with Inversion Recovery (SPIR). A standard gradient echo mDixon water image is shown as the geometrical reference below, along with the B_0_ map for comparison. The red contour outlines the undistorted shape of the anatomy. Large geometric distortions can be seen in the shoulder region of the images without distortion correction (marked by the red arrows). This can be corrected during postprocessing using the estimated B_0_ map. Such B_0_ inhomogeneities can also lead to the failure of SPIR fat‐suppression (marked by the green arrow), which can be avoided using the chemical shift‐encoded MSND approach. FFE, fast field echo (gradient echo)

Figure 8 finally shows that geometric distortions in the MSND results can be corrected in a postprocessing step using the estimated B_0_ map. Furthermore, fat signals that cannot be suppressed by SPIR are effectively removed by chemical shift encoding using the MSND algorithm for reconstruction.

The computation time of the self‐navigation step was about 7 s (initialization + Gauss‐Newton loop: 1 + 6 s) per slice for typical 4‐shot data (eight channels) using the GPU‐based implementation.

## 5 DISCUSSION

One of the major advantages of using chemical shift‐encoding–based DWI^31^, ^32^ is the ability to avoid adverse effects on the water image quality, which is especially critical in diffusion measurements outside the brain. This is mainly caused by the failure of fat suppression techniques resulting from strong B_0_ and/or B_1_^+^ inhomogeneities^25^, ^27^ due to bad shimming conditions. In contrast to spectrally selective fat saturation approaches, ^23^, ^24^ chemical shift encoding can better address the multiline nature of fat and can also minimize potential magnetization transfer^60^ effects caused by off‐resonance irradiation in fat suppression, which helps to avoid any SNR impairment on the water line.

MSND is a novel algorithm proposed in this work to reconstruct chemical shift‐encoded DW ms‐EPI data with improved image quality. In comparison with our previously proposed IDE^32^ method, the MSND algorithm does not require a measured extra navigator, improving sampling efficiency this way, and produces better water/fat‐separated DW images. This can be mainly attributed to the water/fat‐resolved self‐navigation step, which provides a more reliable estimation of the physiological motion‐induced shot‐to‐shot phase variations, while also correcting for fat‐displacement artefacts. To further confirm the impact of the self‐navigation, an intermediate step using the extended model‐based water/fat separation with measured extra navigator was included in Figure 4. Compared with the full MSND reconstruction, similar artefacts as IDE can be seen in the images due to the fat signals present in the navigator data. This also illustrates the importance of the proposed water/fat‐resolved self‐navigation method, as shown in Figure 5. Superior image quality of MSND compared with IDE was shown both in leg, where B_0_ is relatively homogeneous but different fat compositions are encountered (subcutaneous fat and bone marrow), as well as in head–neck images, where relatively large B_0_ inhomogeneities are present. This was supported by statistically significant improvements in CoV, as demonstrated in the leg data in Figure 6, and also the ADC quantifications in both anatomies compared with conventional fat saturation techniques^23^, ^24^ in the supporting information (S.2).

Our presented self‐navigated method overcomes the three major drawbacks of measuring extra 2D navigators for chemical shift‐encoded DWI. First, the acquisition time is prolonged. When the navigator is not measured, each shot duration time can be reduced by 30% to 35%, as shown in Table 1. Second, the SNR of the navigator data is poor because of the long TE (larger than 100 ms). This is even more crucial for measurements outside of the brain, because, for example, muscle tissues exhibit lower T_2_ values (around 32 ms at 3 T^61^). The third drawback is that unsuppressed fat signals, present in the navigator, are shifted with respect to the water along the phase‐encoding direction. These fat‐related artefacts may lead to phase ambiguities and may appear as artifacts in the associated regions of the DW image, as illustrated in Figure 4. By comparison, the self‐navigated MSND can avoid these adverse effects, producing improved DW images, as shown in Figures 4 and 6.

In the proposed MSND algorithm, the water/fat‐resolved self‐navigation step is realized by starting the formulation of the extended signal model directly from the k‐space data and thereby including the fat off‐resonance–related artefact sources in the model. Thus the spatial mismatch between the fat signals in the EPI images and the CSM is automatically corrected during reconstruction. Moreover, similar to the approach of Guo et al.,^11^ we assumed that each shot image has the same magnitude. This helps to better condition the inverse problem and stabilize the “water‐fat merged” phase estimation of the individual shots/chemical shift points. It is noteworthy that k‐space–based water/fat separation has already been explored in many studies,^50^, ^51^, ^62^ which allows for a more accurate correction of the fat off‐resonance effects.

In self‐navigation, the motion‐induced phase errors are represented as shot‐specific phase terms in the signal model (Equation 1). Calculating shot‐to‐shot phase variations while separating water/fat images may lead to the typical water/fat swap artifacts,^42^ because the phase estimation may also lead the optimization to be trapped in suboptimal local minima. Therefore, a reasonable initialization map is important to help prevent inaccurate separation for each pixel and accelerate the convergence. In this particular case, SENSE‐based water/fat separation^43^ was used to calculate water/fat‐separated shot‐to‐shot phase maps. However, when the number of shots increases, the estimation of the shot‐wise phase maps will be compromised because of the reduced conditioning. Therefore, such a water‐fat MUSE algorithm may not be sufficient for use as a stand‐alone self‐navigation method. Nevertheless, it can still be employed as a good initialization for the following iterative phase estimation step, as shown in Figure 3 and in the supporting information (S.4), being especially helpful in the case of low SNR and complex anatomies (e.g., head–neck regions). On the other hand, in such measurements with critical SNR, denoising methods^63^, ^64^, ^65^ may also be needed to further improve the computational stability. This is important, especially when undersampling is considered (meaning subsampling in the phase‐encoding and/or chemical shift‐encoding direction) to further increase scan efficiency, which could become a focus of future work.

In this work, there are a couple of parameters that need to be tuned, for example, stopping criteria for the Gauss‐Newton loop and the internal CG loops, k‐space filter size, and regularization factors of the initialization. This is a drawback of most model‐based reconstruction techniques. However, for most parameters, we found that the same values could be used for all anatomies, volunteers, and scans. An exception is the size of the k‐space window, used to enforce smoothness of the phase maps, which is tuned to a specific resolution and the window's k‐space extent must therefore be adapted for different FOVs. One of the future targets could be to automatically derive the model parameters based on a SNR estimate of the data to further enhance the reconstruction efficiency.

In addition, calibration of the CSM has been shown to be important in this research. In addition to chemical shifts, introducing spatial mismatch between EPI images and CSMs, geometric distortions have a similar effect. Distorted EPI images are encountered with incorrect sensitivity in areas where B_0_ is not homogeneous. In this work, to better illustrate the influence of this mismatch, an experiment was set up by distorting the CSM obtained from the prescan using the B_0_ map, and the result was compared with that using an undistorted CSM. As shown in Figure 2, an additional calibration step is important to avoid the appearance of artefacts. CSM autocalibration from the EPI data itself, using ESPIRiT^52^/J‐SENSE^43^, ^53^, ^56^ or any other methods,^66^ can be more straightforward and easier to apply. In this work, except for the comparison in Figure 2, all datasets were reconstructed with the two‐step autocalibration method. The one‐step autocalibration also shows its feasibility in the leg region in S.3. However, for head–neck regions where B_0_ is more inhomogeneous, the use of regularization^32^, ^41^, ^42^, ^54^ to avoid estimating local minima is necessary and needs further investigation. Moreover, after the whole reconstruction is done, a single postprocessing step^46^, ^59^ is performed to efficiently correct the geometric distortions of the water and/or the fat images (Figure 8). As an alternative, tilted‐CAIPI enhanced PSF‐EPI–based approaches^67^ for DWI can also provide geometric distortion‐free images with water/fat separation resolved.^31^ It would be interesting in the future to combine the proposed self‐navigation method with PSF‐EPI acquisition.

In the proposed reconstruction pipeline, it is the aim to fully leverage all data sampled in a usual DWI scan. Therefore, the b = 0 s/mm^2^ data are used to provide a reference for the DW images in different aspects (e.g., water‐fat masks, calibration of CSM, estimation of B_0_ map). This is a slight limitation of the current reconstruction strategy because it assumes that DW images reflect the same anatomical structure as nondiffusion images (b = 0 s/mm^2^). Correction for potential bulk motion‐induced^16^, ^47^ mismatches or slight diffusion gradient switching‐induced eddy current effects warrant further investigation. This may also enable applications in other regions of the body (e.g., abdomen and liver DWI), where more severe bulk motion‐related issues^68^ occur. Notably, this work focuses more on the performance of the reconstruction. In actual clinical acquisitions, for relatively B_0_ homogeneous regions like legs, it is not necessary to perform 6‐shot scans.

As mentioned above, in this work, the B_0_ map is estimated using the IDE algorithm with b = 0 s/mm^2^ data, which is conventionally acquired for any diffusion measurements at 3 T, to further correct geometric distortion. However, the B_0_ map can also be acquired by a separate prescan.^36^, ^69^, ^70^ With such prior B_0_ information, the use of “two‐point” chemical shift encoding^71^, ^72^ becomes feasible to improve the scan efficiency. Furthermore, compared with the single TE water/fat‐resolved CSM estimation,^43^, ^44^ the proposed pipeline with the additional chemical shift‐encoding dimension allows the MSND algorithm to jointly estimate the CSM and the B_0_ map from the b = 0 s/mm^2^ data, as shown in the supporting information (S.3). In the current strategy, B_0_ estimation is not repeated for each b‐value when reconstructing the DW images using the MSND algorithm. The MSND algorithm is assumed to be able to potentially correct for slight B_0_ variations (e.g., caused by eddy currents when switching the strong diffusion gradients),^73^, ^74^ which could potentially be treated as a smooth phase term for each gradient direction and could be captured by the self‐navigation process. However, currently this should still be considered as speculation: future experiments will have to confirm this.

## 6 CONCLUSION

A new algorithm (MSND) is established to enable improved water/fat separation based on chemical shift‐encoded DW ms‐EPI, while removing the shot‐to‐shot physiological motion‐induced phase variations using a self‐navigation approach. In vivo experiments showed that this approach improves water‐only DW images compared with other reference algorithms while increasing the DWI sampling efficiency at the same time.

## Supporting information


**Data S1.** Supporting Information

## References

[1] Basser PJ , Mattiello J , LeBihan D . MR diffusion tensor spectroscopy and imaging. Biophys J. 1994;66(1):259‐267. doi:10.1016/S0006-3495(94)80775-1 8130344 PMC1275686

[2] Connolly M , Srinivasan A . Diffusion‐weighted imaging in head and neck cancer: technique, limitations, and applications. Magn Reson Imaging Clin Am. 2018;26(1):121133. doi:10.1016/j.mric.2017.08.011 29128000

[3] le Bihan D , Mangin JF , Poupon C , et al. Diffusion tensor imaging: Concepts and applications. J Magn Reson Imaging. 2001;13(4):534546 doi:10.1002/jmri.1076 11276097

[4] Jezzard P , Balaban RS . Correction for geometric distortion in echo planar images from B0 field variations. Magn Reson Med. 1995;34(1):65‐73. doi:10.1002/mrm.1910340111 7674900

[5] McKinnon GC . Ultrafast interleaved gradient‐echo‐planar imaging on a standard scanner. Magn Reson Med. 1993;30(5):609‐616. doi:10.1002/mrm.1910300512 8259061

[6] Skare S , Newbould RD , Clayton DB , Albers GW , Nagle S , Bammer R . Clinical multishot DW‐EPI through parallel imaging with considerations of susceptibility, motion, and noise. Magn Reson Med. 2007;57(5):881‐890. doi:10.1002/mrm.21176 17457876 PMC3986024

[7] Jeong HK , Gore JC , Anderson AW . High‐resolution human diffusion tensor imaging using 2‐D navigated multishot SENSE EPI at 7 T. Magn Reson Med. 2013;69(3):793‐802. doi:10.1002/mrm.24320 22592941 PMC3424313

[8] O'Halloran RL , Holdsworth S , Aksoy M , Bammer R . Model for the correction of motion‐induced phase errors in multishot diffusion‐weighted‐MRI of the head: are cardiac‐motion‐induced phase errors reproducible from beat‐to‐beat? Magn Reson Med. 2012;68(2):430‐440. doi:10.1002/mrm.23245 22213138 PMC3320700

[9] Anderson AW , Gore JC . Analysis and correction of motion artifacts in diffusion weighted imaging. Magn Reson Med. 1994;32(3):379‐387. doi:10.1002/mrm.1910320313 7984070

[10] Miller KL , Pauly JM . Nonlinear phase correction for navigated diffusion imaging. Published online 2003. 10.1002/mrm.10531 12876711

[11] Guo H , Ma X , Zhang Z , Zhang B , Yuan C , Huang F . POCS‐enhanced inherent correction of motion‐induced phase errors (POCS‐ICE) for high‐resolution multishot diffusion MRI. Magn Reson Med. 2016;75(1):169‐180. doi:10.1002/mrm.25594 25648591

[12] Butts K , de Crespigny A , Pauly JM , Moseley M . Diffusion‐weighted interleaved echo‐planar imaging with a pair of orthogonal navigator echoes. Magn Reson Med. 1996;35(5):763‐770. doi:10.1002/mrm.1910350518 8722828

[13] Bammer R , Stollberger R , Augustin M , et al. Diffusion‐weighted imaging with navigated interleaved echo‐planar imaging and a conventional gradient system. Radiology. 1999;211(3):799‐806. doi:10.1148/radiology.211.3.r99jn15799 10352609

[14] Truong TK , Guidon A . High‐resolution multishot spiral diffusion tensor imaging with inherent correction of motion‐induced phase errors. Magn Reson Med. 2014;71(2):790‐796. doi:10.1002/mrm.24709 23450457 PMC3949176

[15] Chen NK , Guidon A , Chang HC , Song AW . A robust multi‐shot scan strategy for high‐resolution diffusion weighted MRI enabled by multiplexed sensitivity‐encoding (MUSE). NeuroIimage. 2013;72:41‐47. doi:10.1016/j.neuroimage.2013.01.038 PMC3602151 23370063

[16] Steinhoff M , Nehrke K , Mertins A , Börnert P . Segmented diffusion imaging with iterative motion‐corrected reconstruction (SEDIMENT) for brain echo‐planar imaging. NMR Biomed. 2020;33(12):e4185. doi:10.1002/nbm.4185 31814181

[17] Moeller S , Ramanna S , Lenglet C , et al. Self‐navigation for 3D multishot EPI with data‐reference. Magn Reson Med. 2020;84(4):1747‐1762. doi:10.1002/mrm.28231 32115756 PMC7329618

[18] Hu Z , Ma X , Truong TK , Song AW , Guo H . Phase‐updated regularized SENSE for navigator‐free multishot diffusion imaging. Magn Reson Med. 2017;78(1):172‐181. doi:10.1002/mrm.26361 27520840

[19] Mani M , Aggarwal HK , Magnotta V , Jacob M . Improved MUSSELS reconstruction for high‐resolution multi‐shot diffusion weighted imaging. Magn Reson Med. 2020;83(6):2253‐2263. doi:10.1002/mrm.28090 31789440 PMC8045517

[20] Mani M , Jacob M , Kelley D , Magnotta V . Multi‐shot sensitivity‐encoded diffusion data recovery using structured low‐rank matrix completion (MUSSELS). Magn Reson Med. 2017;78(2):494‐507. doi:10.1002/mrm.26382 27550212 PMC5336529

[21] Hu Y , Levine EG , Tian Q , et al. Motion‐robust reconstruction of multishot diffusion‐weighted images without phase estimation through locally low‐rank regularization. Magn Reson Med. 2019;81(2):1181‐1190. doi:10.1002/mrm.27488 30346058 PMC6289606

[22] Khalek AA , Nermin AR , Soliman Y , Elkhamary S , Alsharaway MK , Tawfik A . Head and neck role of diffusion‐weighted MR imaging in cervical lymphadenopathy. Eur Radiol. 2006;16:1468‐1477. doi:10.1007/s00330-005-0133-x 16557366

[23] Zee CS , Segall HD , Terk MR , et al. SPIR MRI in spinal diseases. J Comput Assist Tomogr. 1992;16(3):356‐360. doi:10.1097/00004728-199205000-00004 1592915

[24] Udayasankar UK , Martin D , Lauenstein T , et al. Role of spectral presaturation attenuated inversion‐recovery fat‐suppressed T2‐weighted MR imaging in active inflammatory bowel disease. J Magn Reson Imaging. 2008;28(5):1133‐1140. doi:10.1002/jmri.21574 18972354

[25] Wendl CM , Eiglsperger J , Dendl LM , et al. Fat suppression in magnetic resonance imaging of the head and neck region: Is the two‐point DIXON technique superior to spectral fat suppression? Br J Radiol. 2018;91(1085):20170078. doi:10.1259/bjr.20170078 29436841 PMC6190774

[26] Bae YJ , Choi BS , Jeong HK , Sunwoo L , Jung C , Kim JH . Diffusion‐weighted imaging of the head and neck: Influence of fat‐suppression technique and multishot 2D navigated interleaved acquisitions. Am J Neuroradiol. 2018;39(1):145‐150. doi:10.3174/ajnr.A5426 29122759 PMC7410699

[27] Anzai Y , Lufkin RB , Jabour BA , Hanafee WN . Fat‐suppression failure artifacts simulating pathology on frequency‐selective fat‐suppression MR images of the head and neck. AJNR Am J Neuroradiol. 1992;13(3):879‐884. 1590186 PMC8331697

[28] Burakiewicz J , Charles‐Edwards GD , Goh V , Schaeffter T . Water‐fat separation in diffusion‐weighted EPI using an IDEAL approach with image navigator. Magn Reson Med. 2015;73(3):964‐972. doi:10.1002/mrm.25191 24723244

[29] Burakiewicz J , Hooijmans MT , Webb AG , Verschuuren JJGM , Niks EH , Kan HE . Improved olefinic fat suppression in skeletal muscle DTI using a magnitude‐based dixon method. Magn Reson Med. 2018;79(1):152‐159. doi:10.1002/mrm.26655 28261865

[30] Hernando D , Karampinos DC , King KF , et al. Removal of olefinic fat chemical shift artifact in diffusion MRI. Magn Reson Med. 2011;65(3):692‐701. doi:10.1002/mrm.22670 21337402 PMC3069507

[31] Hu Z , Wang Y , Dong Z , Guo H . Water/fat separation for distortion‐free EPI with point spread function encoding. Magn Reson Med. 2019;82(1):251‐262. doi:10.1002/mrm.27717 30847991

[32] Dong Y , Koolstra K , Riedel M , van Osch MJP , Börnert P . Regularized joint water–fat separation with B0 map estimation in image space for 2D‐navigated interleaved EPI based diffusion MRI. Magn Reson Med. 2021;86(6):3034‐3051. doi:10.1002/mrm.28919 34255392 PMC8596522

[33] Ren J , Dimitrov I , Sherry AD , Malloy CR . Composition of adipose tissue and marrow fat in humans by 1H NMR at 7 Tesla. J Lipid Res. 2008;49(9):2055‐2062. doi:10.1194/jlr.D800010-JLR200 18509197 PMC2515528

[34] Hamilton G , Yokoo T , Bydder M , et al. In vivo characterization of the liver fat 1H MR spectrum. NMR Biomed. 2011;24(7):784‐790. doi:10.1002/nbm.1622 21834002 PMC3860876

[35] Glover GH , Schneider E . Three‐point Dixon technique for true water/fat decomposition with B0 inhomogeneity correction. Magn Reson Med. 1991;18(2):371‐383. doi:10.1002/mrm.1910180211 2046518

[36] Reeder SB , Wen Z , Yu H , et al. Multicoil Dixon chemical species separation with an iterative least‐squares estimation method. Magn Reson Med. 2004;51(1):35‐45. doi:10.1002/mrm.10675 14705043

[37] Eggers H , Börnert P . Chemical shift encoding‐based water‐fat separation methods. J Magn Reson Imaging. 2014;40(2):251‐268. doi:10.1002/jmri.24568 24446249

[38] Robson MD , Gore JC , Constable RT . Measurement of the point spread function in MRI using constant time imaging. Magn Reson Med. 1997;38(5):733‐740. doi:10.1002/mrm.1910380509 9358447

[39] Zaitsev M , Hennig J , Speck O . Point spread function mapping with parallel imaging techniques and high acceleration factors: Fast, robust, and flexible method for echo‐planar imaging distortion correction. Magn Reson Med. 2004;52(5):1156‐1166. doi:10.1002/mrm.20261 15508146

[40] Pruessmann KP , Weiger M , Scheidegger MB , Boesiger P . SENSE: Sensitivity encoding for fast MRI. Magn Reson Med. 1999;42(5):952‐962. doi:10.1002/(SICI)1522‐2594(199911)42:5<952::AID‐MRM16>3.0.CO;2‐S 10542355

[41] Hernando D , Kellman P , Haldar JP , Liang ZP . Robust water/fat separation in the presence of large field inhomogeneities using a graph cut algorithm. Magn Reson Med. 2010;63(1):79‐90. doi:10.1002/mrm.22177 19859956 PMC3414226

[42] Yu H , Reeder SB , Shimakawa A , Brittain JH , Pelc NJ . Field map estimation with a region growing scheme for iterative 3‐point water‐fat decomposition. Magn Reson Med. 2005;54(4):1032‐1039. doi:10.1002/mrm.20654 16142718

[43] Uecker M . Making SENSE of chemical shift: separating species in single‐shot EPI using multiple coils. ISMRM. 2012;20:2490.

[44] Shin PJ , Larson PEZ , Uecker M , et al. Chemical shift separation with controlled aliasing for hyperpolarized 13C metabolic imaging. Magn Reson Med. 2015;74(4):978‐989. doi:10.1002/mrm.25473 25298086 PMC4390401

[45] Seginer A , Furman‐Haran E , Goldberg I , Schmidt R . Reducing SAR in 7T brain fMRI by circumventing fat suppression while removing the lipid signal through a parallel acquisition approach. Sci Rep. 2021;11(1):15371. doi:10.1038/s41598-021-94692-6 34321529 PMC8319205

[46] Koolstra K , O'Reilly T , Börnert P , Webb A . Image distortion correction for MRI in low field permanent magnet systems with strong B0 inhomogeneity and gradient field nonlinearities. Magn Reson Mater Phys Biol Med. 2021;34(4):631‐642. doi:10.1007/s10334-021-00907-2 PMC8338849 33502668

[47] Guhaniyogi S , Chu ML , Chang HC , Song AW , Chen NK . Motion immune diffusion imaging using augmented MUSE for high‐resolution multi‐shot EPI. Magn Reson Med. 2016;75(2):639‐652. doi:10.1002/mrm.25624 25762216 PMC4567534

[48] Munger P , Greller GR , Peters TM , Pike GB . An inverse problem approach to the correction of distortion in EPI images. IEEE Trans Med Imaging. 2000;19(7):681‐689. doi:10.1109/42.875186 11055783

[49] Andersson JLR , Skare S , Ashburner J . How to correct susceptibility distortions in spin‐echo echo‐planar images: Application to diffusion tensor imaging. NeuroIimage. 2003;20(2):870‐888. doi:10.1016/S1053-8119(03)00336-7 14568458

[50] Honorato JL , Parot V , Tejos C , Uribe S , Irarrazaval P . Chemical species separation with simultaneous estimation of field map and T2* using a k‐space formulation. Magn Reson Med. 2012;68(2):400‐408. doi:10.1002/mrm.23237 22212998

[51] Berglund J , Rydén H , Avventi E , Norbeck O , Sprenger T , Skare S . Fat/water separation in k‐space with real‐valued estimates and its combination with POCS. Magn Reson Med. 2020;83(2):653‐661. doi:10.1002/mrm.27949 31418932

[52] Uecker M , Lai P , Murphy MJ , et al. ESPIRiT ‐ An eigenvalue approach to autocalibrating parallel MRI: Where SENSE meets GRAPPA. Magn Reson Med. 2014;71(3):990‐1001. doi:10.1002/mrm.24751 23649942 PMC4142121

[53] Uecker M , Hohage T , Block KT , Frahm J . Image reconstruction by regularized nonlinear inversion ‐ Joint estimation of coil sensitivities and image content. Magn Reson Med. 2008;60(3):674‐682. doi:10.1002/mrm.21691 18683237

[54] Lu W , Hargreaves BA . Multiresolution field map estimation using golden section search for water‐fat separation. Magn Reson Med. 2008;60(1):236‐244. doi:10.1002/mrm.21544 18581397

[55] Liu C , Bammer R , Moseley ME . Parallel imaging reconstruction for arbitrary trajectories using k‐space sparse matrices (kSPA). Magn Reson Med. 2007;58(6):1171‐1181. doi:10.1002/mrm.21334 17969012 PMC3985775

[56] Ying L , Sheng J . Joint image reconstruction and sensitivity estimation in SENSE (JSENSE). Magn Reson Med. 2007;57(6):1196‐1202. doi:10.1002/mrm.21245 17534910

[57] Ong F , Lustig M. SigPy: A Python Package for High Performance Iterative Reconstruction. ISMRM. 2019. Accessed January 10, 2022. https://archive.ismrm.org/2019/4819.html

[58] Huang F , Vijayakumar S , Li Y , Hertel S , Duensing GR . A software channel compression technique for faster reconstruction with many channels. Magn Reson Imaging. 2008;26(1):133‐141. doi:10.1016/j.mri.2007.04.010 17573223

[59] Man LC , Pauly JM , Macovski A . Multifrequency interpolation for fast off‐resonance correction. Magn Reson Med. 1997;37(5):785‐792. doi:10.1002/mrm.1910370523 9126954

[60] Henkelman RM , Stanisz GJ , Graham SJ . Magnetization transfer in MRI: a review. NMR Biomed. 2001;14(2):57‐64. doi:10.1002/nbm.683 11320533

[61] Gold GE , Han E , Stainsby J , Wright G , Brittain J , Beaulieu C . Musculoskeletal MRI at 3.0 T: relaxation times and image contrast. Am J Roentgenol. 2004;183(2):343‐351. doi:10.2214/ajr.183.2.1830343 15269023

[62] Brodsky EK , Holmes JH , Yu H , Reeder SB . Generalized K‐space decomposition with chemical shift correction for non‐Cartesian water‐fat imaging. Magn Reson Med. 2008;59(5):1151‐1164. doi:10.1002/mrm.21580 18429018

[63] Fadnavis S , Batson J , Garyfallidis E . Patch2Self: denoising diffusion MRI with self‐supervised learning. Adv Neural Inf Process Syst. 2020;33:16293‐16303.

[64] Veraart J , Novikov DS , Christiaens D , Ades‐aron B , Sijbers J , Fieremans E . Denoising of diffusion MRI using random matrix theory. NeuroIimage. 2016;142:394‐406. doi:10.1016/j.neuroimage.2016.08.016 PMC5159209 27523449

[65] Tian Q , Li Z , Fan Q , et al. SDnDTI: Self‐supervised deep learning‐based denoising for diffusion tensor MRI. NeuroIimage. 2022;253:119033. doi:10.1016/j.neuroimage.2022.119033 PMC9511973 35240299

[66] Peng X , Sutton BP , Lam F , Liang ZP . DeepSENSE: Learning coil sensitivity functions for SENSE reconstruction using deep learning. Magn Reson Med. 2022;87(4):1894‐1902. doi:10.1002/mrm.29085 34825732 PMC8810692

[67] Dong Z , Wang F , Reese TG , et al. Tilted‐CAIPI for highly accelerated distortion‐free EPI with point spread function (PSF) encoding. Magn Reson Med. 2019;81(1):377‐392. doi:10.1002/mrm.27413 30229562 PMC6258292

[68] Stemkens B , Benkert T , Chandarana H , et al. Adaptive bulk motion exclusion for improved robustness of abdominal magnetic resonance imaging. NMR Biomed. 2017;30(11):e3830. 28885742 10.1002/nbm.3830 PMC5643254

[69] Robinson S , Jovicich J . B0 mapping with multi‐channel RF coils at high field. Magn Reson Med. 2011;66(4):976‐988. doi:10.1002/mrm.22879 21608027

[70] Funai AK , Fessler JA , Yeo DTB , Noll DC , Olafsson VT . Regularized field map estimation in MRI. IEEE Trans Med Imaging. 2008;27(10):1484‐1494. doi:10.1109/TMI.2008.923956 18815100 PMC2856353

[71] Eggers H , Brendel B , Duijndam A , Herigault G . Dual‐echo Dixon imaging with flexible choice of echo times. Magn Reson Med. 2011;65(1):96‐107. doi:10.1002/mrm.22578 20860006

[72] Berglund J , Ahlström H , Johansson L , Kullberg J . Two‐point Dixon method with flexible echo times. Magn Reson Med. 2011;65(4):994‐1004. doi:10.1002/mrm.22679 21413063

[73] Alhamud A , Taylor PA , van der Kouwe AJW , Meintjes EM . Real‐time measurement and correction of both B0 changes and subject motion in diffusion tensor imaging using a double volumetric navigated (DvNav) sequence HHS public sccess. NeuroIimage. 2016;126:60‐71. doi:10.1016/j.neuroimage.2015.11.022 PMC4733594 26584865

[74] Benner T , van der Kouwe AJW , Kirsch JE , Sorensen AG . Real‐time RF pulse adjustment for Bo drift correction. Magn Reson Med. 2006;56(1):204‐209. doi:10.1002/mrm.20936 16767763

[75] Pokharel SS , Macura KJ , Kamel IR , Zaheer A . Current MR imaging lipid detection techniques for diagnosis of lesions in the abdomen and pelvis. Radiographics. 2013;33(3):681‐702. doi:10.1148/rg.333125068 23674769

[76] Block KT , Uecker M , Frahm J . Undersampled radial MRI with multiple coils. Iterative image reconstruction using a total variation constraint. Magn Reson Med. 2007;57(6):1086‐1098. doi:10.1002/mrm.21236 17534903

