# Supplementary material for: Water/fat separation for self‐navigated diffusion‐weighted multishot echo‐planar imaging
Source: NMR Biomed. 2022 Sep 13;36(1):e4822. doi: 10.1002/nbm.4822 (PMC10078174; doi:10.1002/nbm.4822)
Supplement: Supplementary file 1 — Data S1. Supporting Information [file NBM-36-0-s001.docx]

**Supporting information**

**S.1.** Linear operators

An appropriate way to implement the proposed approach is to make use of linear operators instead of constructing a dense system matrix. The main advantage of such an approach is the much lower memory requirement and that one can make use of the efficient implementation of the FFT operator. The coefficient matrix $\hat{A}$ can simply be implemented by multiplying all the operators in Eq.3. In the following, each individual operator in the forward model Eq.3 is described in more detail. To clearly show the forward modeling, the description of Eq. 3 is carried out from right to left. Please note that, for real implementation, if the linear operators are used, the backward model may need also to be constructed correspondingly, using operators as described above.

Assuming a data set with $N$ chemical shift encoding steps, $L$ shots, $J$ coils, and $Q$ pixels for each water/fat image at a given b-value, each linear operator in the forward model can be expressed as:

- Diffusion phase operator $\hat{\Phi}$ ($N\times L\times Q,Q$). The phase map $\Phi_{n,l}$ for each individual chemical shift point $n$ and shot $l$ can be acquired through either measuring an extra 2D-navigator^6^ or by performing the proposed self-navigation described in the theory chapter. This operator will add a phase modulation term for both pure water and fat images as $\rho_{w}\left( r \right)e^{-i\phi_{n,l}\left( r \right)}$ and $\rho_{f}\left( r \right)e^{-i\phi_{n,l}\left( r \right)}$ for each shot $l$, chemical shift point $n$ and pixel $r$.
- B_0_ map operator $\hat{\Psi}_{B}$ ($N\times L\times Q,N\times L\times Q$). This operator modulates the B_0_ phase term for water and fat in image space, assuming a constant time map for the EPI data at each $\Delta TE$ point (for demodulating B_0_ in k-space, see Supporting Information S.3). This operator will not change the dimensions of the data at this step, but will add different B_0_ phase modulation for each chemical shift point $n$, as $\rho_{w}\left( r \right)e^{-i2\pi\psi_{B}\left( r \right)\Delta{TE}_{n}}e^{-i\phi_{n,l}\left( r \right)}$ and $\rho_{f}\left( r \right)e^{-i2\pi\psi_{B}\left( r \right)\Delta{TE}_{n}}e^{-i\phi_{n,l}\left( r \right)}$.
- SENSE operator $\hat{C}$ ($J\times N\times L\times Q,N\times L\times Q$). One of the main benefits of using the extended full model is the ability to correct the spatial mismatch between the CSM data, measured with a short TE gradient echo, and EPI data caused by off-resonance effects. At this moment the only change to the data are the added sensitivity weights for each coil $j$, which is the same for water/fat as $c_{j}\left( r \right)\rho_{w}\left( r \right)e^{-i2\pi\psi_{B}\left( r \right)\Delta{TE}_{n}}e^{-i\phi_{n,l}\left( r \right)}$ and $c_{j}\left( r \right)\rho_{f}\left( r \right)e^{-i2\pi\psi_{B}\left( r \right)\Delta{TE}_{n}}e^{-i\phi_{n,l}\left( r \right)}$.
- Fourier operator $\hat{F}$ ($J\times N\times L\times Q,J\times N\times L\times Q$) to perform the Fourier transform on water/fat images, respectively. The signals of water and fat will become $\int c_{j}\left( r \right)\rho_{w}\left( r \right)e^{-i2\pi\psi_{B}\left( r \right)\Delta{TE}_{n}}e^{-i\phi_{n,l}\left( r \right)}e^{-ik_{t}\cdot r}dr$ and $\int c_{j}\left( r \right)\rho_{f}\left( r \right)e^{-i2\pi\psi_{B}\left( r \right)\Delta{TE}_{n}}e^{-i\phi_{n,l}\left( r \right)}e^{-ik_{t}\cdot r}dr$.
- Fat off-resonance operator $\hat{\Psi}_{f}$ ($J\times N\times L\times Q,J\times N\times L\times Q$). This operator acts only on the fat channel and introduces fat peak-specific off-resonances into the forward model. Considering the multi-peak spectral nature of fat, an $M$-peak model is employed. In contrast to modulating fat off-resonance in image space, modelling in k-space allows correction of chemical shift effects in both the readout and phase encoding direction. Please be noticed that, after merging the water and fat channels of the signals with the operation $\left[ \hat{I} \hat{I} \right]$ in Eq. 3, the whole signal model will be the one as Eq.1.
- Shot sampling operator $\hat{K}$ with operator matrix shape ($J\times N\times L\times Q/L,J\times N\times L\times Q$). This operator defines the sampling patten for each shot with the corresponding phase encoding sampling lines in k-space. This will divide the signals into different shots to form the actual ms-EPI data.

**S.2.** Comparison of ADC mapping between MSND and fat saturation techniques.


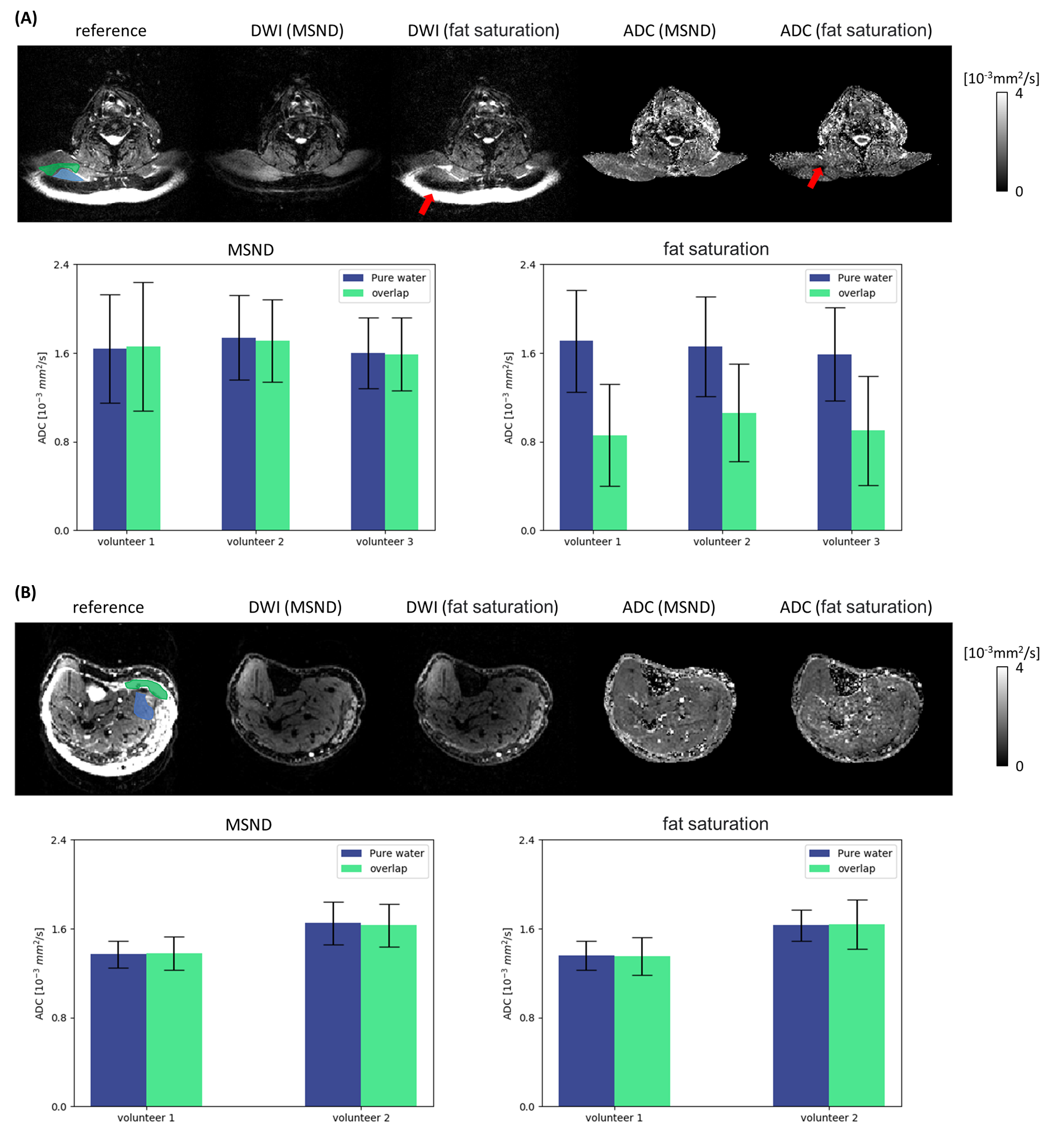


ADC mapping evaluations were conducted in two legs (where B_0_ is relatively homogeneous) and three shoulder slices (where B_0_ is inhomogeneous) from 5 different volunteers. The MSND reconstruction was compared to the data using spectrally selective fat saturation for the same slice, shown with the reference (b=0 s/mm^2^_,_ reconstructed through conventional shot-combination and SENSE of one chemical-shift encoding step), diffusion images of both methods (b=300 s/mm^2^), and ADC maps of both methods. SPIR fat suppression was used for the shoulder scans, while SPAIR was used for the leg to avoid potential B_1_^+^ inhomogeneity-induced issues^32,75^. Two ROIs were selected for each dataset as pure water regions (marked in blue) and water-fat overlapping regions (marked in green). Both regions belong to the same muscle, therefore similar water ADC values are expected. Paired t-tests were performed between the two ROIs of each method/slice to evaluate the differences in the ADC values between the two regions. In the leg data, the ADC obtained with MSND shows comparable values as SPIR in both ROIs. The large P-values between two ROIs for the two slices were 0.61/0.48 of MSND, and 0.70/0.54 of SPAIR, indicating no significant differences for MSND and for SPAIR (P > 0.05).

In the shoulder slices, where fat suppression failed due to B_0_ inhomogeneities, the ADC values are comparable in the pure water regions for MSND and SPIR results, but distinctly different in the water-fat overlapping regions. For MSND, the calculated P-values between two ROIs of three slices were 0.64/0.88/0.52, indicating no significant differences (P > 0.05). For SPIR data all three slices show small P-values (P<0.05), indicating significantly different ADC quantifications due to the unsuppressed fat signals present. This evaluation illustrates the importance of using chemical shift encoding methods for ADC quantifications especially in the B_0_ inhomogeneous regions.

**S.3.** one-step joint CSM autocalibration with B_0_ map estimation


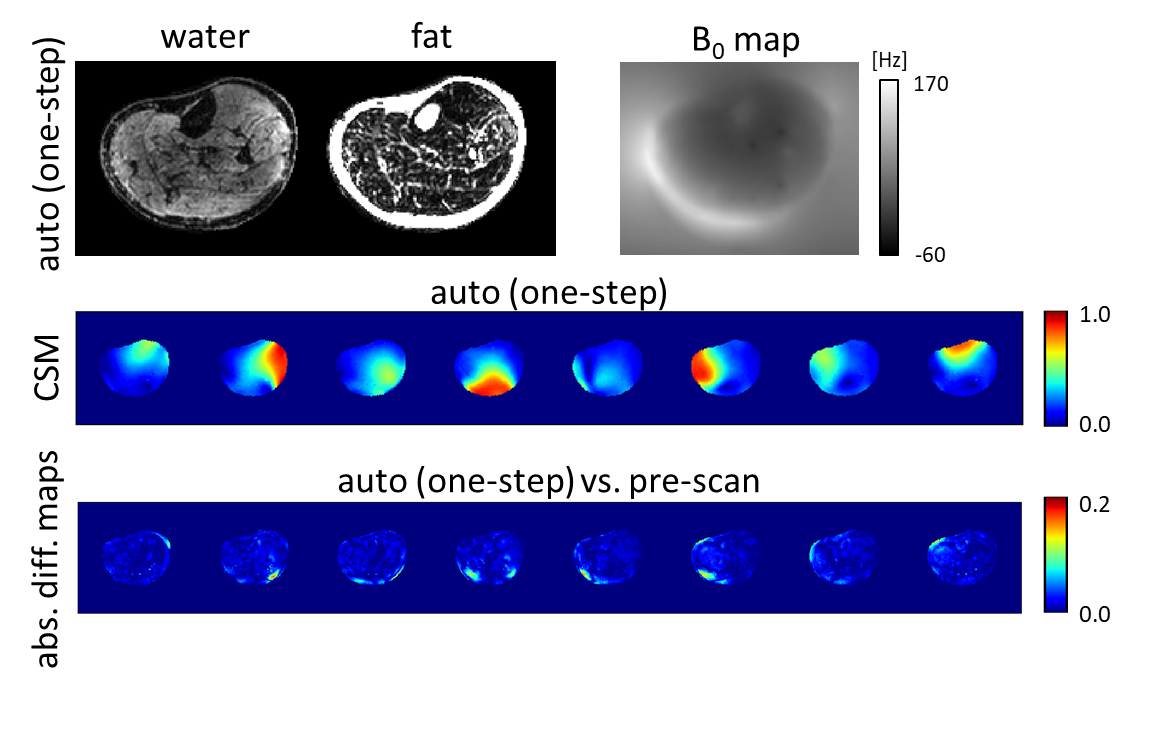


The same DW data (b=600 s/mm^2^) as in Figure 2 of one subject’s leg is shown as additional comparison for one-step calibration method. The CSM and the B_0_ map are estimated jointly from b=0 s/mm^2^ data and used to reconstruct the DW water/fat images. Two TV regularization terms are used for B_0_ map^32^ and CSM^76^ to enforce the smoothness, respectively. Compared with Figure 2, the one-step calibration shows comparable results for water/fat separation, B_0_ estimation and CSM calibration to the two-step autocalibration (IDE + ESPIRiT).

**S.4.** Impact of the initialization of the diffusion phase maps when using self-navigation


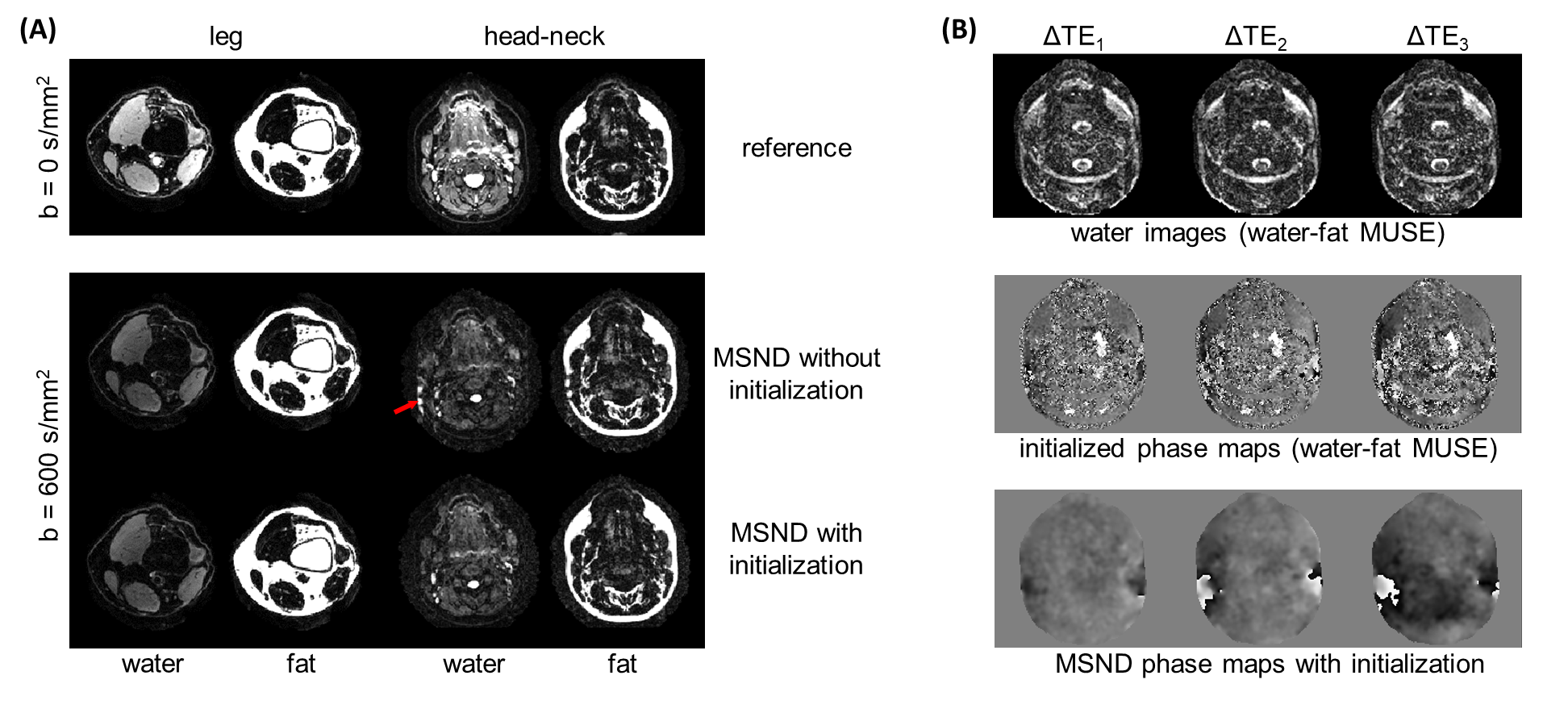


(A) MSND reconstructions of a leg and a head-neck slice (6-shot) are shown with/without initialization by water-fat MUSE. For each data set, a b=0 s/mm^2^ reconstruction is shown as structural reference (given in the top row). The images below show diffusion weighted water/fat separated MSND images using different initializations of the diffusion phase maps. Some swap artefacts can be seen in the head-neck DWI result when no initialization is done (marked by the red arrow, bottom row). This can be avoided by applying water-fat MUSE initialization (middle row). In the more B_0_ homogeneous leg regions, MUSE initialization does not make a significant difference compared to without initialization (zero-phase).

(B) Water-fat MUSE results of the same head-neck slice in S.4(A). Only the first shots of the three chemical-shift encoding steps (6-shot data) are shown. In the top row separated water magnitude images are presented. In the middle row the initial phase maps for initialization are shown, whereas in the bottom row the final MSND estimated phase maps are given (the phase maps are scaled the same). The SENSE based water/fat separation (top row) has been proven effective in several recent publications^43–45^. However, using it directly for DW ms-EPI might be challenging, especially when considering self-navigation. Inspired by MUSE^14^, water-fat SENSE can also be modified as a MUSE-like self-navigation approach. This can be done by treating each individual DW shot data as an under-sampled k-space dataset. The individual shot data can therefore be reconstructed using “water-fat SENSE” instead of the conventional SENSE in MUSE, getting rid of the chemical shift effects of fat. Nevertheless, this approach can also be problematic for high numbers of shots (virtually with a doubled reduction factor due to fat) and cannot be directly used as a robust solution as shown in the first row. However, it still has potential to provide a good initialization (second row) for the following phase estimation step using the Gauss-Newton loop (third row).
